# Review and recommendations for using artificial intelligence in intracoronary optical coherence tomography analysis

**DOI:** 10.1093/ehjdh/ztaf053

**Published:** 2025-05-15

**Authors:** Xu Chen, Yuan Huang, Benn Jessney, Jason Sangha, Sophie Gu, Carola-Bibiane Schönlieb, Martin Bennett, Michael Roberts

**Affiliations:** Department of Medicine, University of Cambridge, Puddicombe Way, Cambridge CB2 0AW, UK; Department of Medicine, University of Cambridge, Puddicombe Way, Cambridge CB2 0AW, UK; Department of Applied Mathematics and Theoretical Physics, University of Cambridge, Cambridge, UK; Department of Medicine, University of Cambridge, Puddicombe Way, Cambridge CB2 0AW, UK; Department of Medicine, University of Cambridge, Puddicombe Way, Cambridge CB2 0AW, UK; Department of Medicine, University of Cambridge, Puddicombe Way, Cambridge CB2 0AW, UK; Department of Applied Mathematics and Theoretical Physics, University of Cambridge, Cambridge, UK; Department of Medicine, University of Cambridge, Puddicombe Way, Cambridge CB2 0AW, UK; Department of Medicine, University of Cambridge, Puddicombe Way, Cambridge CB2 0AW, UK; Department of Applied Mathematics and Theoretical Physics, University of Cambridge, Cambridge, UK

**Keywords:** Optical coherance tomography, Intravascular imaging, Artificial intelligence, Deep learning, Machine learning

## Abstract

Artificial intelligence (AI) tools hold great promise for the rapid and accurate diagnosis of coronary artery disease (CAD) from intravascular optical coherent tomography (IVOCT) images. Numerous papers have been published describing AI-based models for different diagnostic tasks, yet it remains unclear, which models have potential clinical utility and have been properly validated. This systematic review considered published literature between January 2015 and December 2024 describing AI-based diagnosis of CAD using IVOCT. Our search identified 8600 studies, with 629 included after initial screening and 39 studies included in the final systematic review after quality screening. Our findings indicate that most of the identified models are not currently suitable for clinical use, primarily due to methodological flaws and underlying biases. To address these issues, we provide recommendations to improve model quality and research practices to enhance the development of clinically useful AI products.

## Graphical Abstract

**Graphical Abstract:**
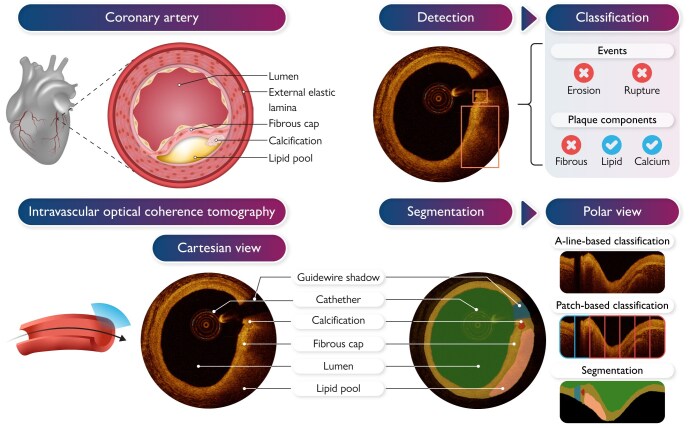

## Introduction

Intravascular optical coherence tomography (IVOCT) is a widely used catheter-based imaging modality utilizing near-infrared light, capable of providing high-resolution cross-sectional images of both coronary arteries and atherosclerotic lesions.^1,2^ IVOCT can identify different tissues including normal vessel, lipid, and calcium, and its superior axial resolution (∼10 μm) compared with other forms of coronary imaging (such as intravascular ultrasound (∼150 μm) and angiography (∼600 μm)) make it particularly useful for assessment of plaque composition, such as lipid and calcium arc as well as micron-level features such as fibrous cap thickness.^3,4^ These properties mean that OCT can help guide percutaneous coronary intervention (PCI),^5^ identify lesions at higher risk of causing future events,^6^ and monitor the efficacy of drugs that may reduce atherosclerotic plaque size or promote plaque stability.^7^ However, as a percentage of all PCI cases, routine IVOCT usage is low. Reasons for this are complex, with a key factor being that IVOCT datasets are large with multiple candidate measurements per artery. Detailed IVOCT analysis requires time-consuming expert clinician or core laboratory analysis, whilst artefacts and limited sampling markedly impair reproducibility.^8–12^ There is a growing demand for automated methods of IVOCT image analysis to address these challenges.

**Figure 1:**
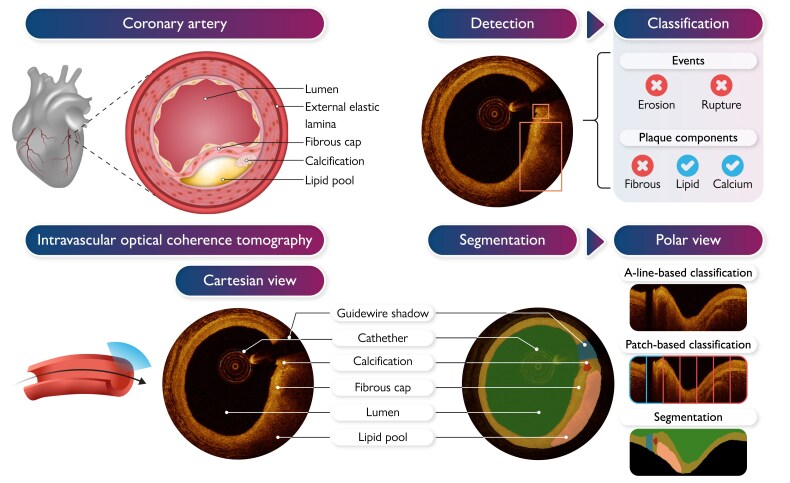
An overview of coronary artery and plaque anatomy, corresponding Cartesian intravascular optical coherent tomography (IVOCT) image, IVOCT detection and segmentation, and use to predict pathology underlying events, measure plaque components, and classify plaques in the polar view.

Artificial intelligence (AI) is a transformative technology that holds great promise to aid or replace humans in completing routine tasks. AI-based models are trained to recognize patterns in data by analysing large datasets and adjusting their internal parameters through an optimization process. As the primary method for developing AI systems, machine learning (ML) requires real-world examples of input and output data to train models that automatically identify the patterns in the data and are able to make predictions based on new input data, e.g. we could train an image segmentation model with images as inputs and segmentation masks as outputs. Traditional ML approaches rely on extracting pre-defined features from data and training models to associate those features to outputs, whereas deep learning (DL)-based approaches can ingest the data directly, automatically extracting highly complex patterns in the data and associating with outcomes. DL approaches are end-to-end, allowing for the automated identification of, potentially very abstract, features which are most useful for the outcome prediction. DL algorithms have demonstrated excellent performance in image processing with several studies using these techniques to segment structures such as the prostate gland in MRI scans^13,14^ and deep brain structures in CT scans,^15^ as well as cancer diagnosis,^16–18^ classification of patient groups,^19^ tumour segmentation,^20^ treatment planning,^21^ and coronary artery disease (CAD) diagnostics^22,23^ (*Figure 1*). AI-based IVOCT analysis may aid both interpretation and patient management. While early studies show promise it is not clear why so few studies achieve clinical translation, with the clear exception of Ultreon (Abbott Laboratories).

Use of IVOCT is proven to improve patient outcomes, through better guiding of stenting and disease tracking, thereby reducing both heart attacks and death for a range of disease states.^24,25^ Therefore, it is important that AI-based tools have the highest possibility of clinical translation. However, noting the increasing^26–28^ concern about the datasets used to train ML models, their validation, reproducibility and generalizability, we explored the current literature with the aim of improving the quality of future AI-based IVOCT assessment through understanding the development methodologies. We found 172 papers using AI/ML methods have been applied to IVOCT, but there was inconsistent standardization such as data collection/selection procedures, performance validation and evaluation metrics. While previous reviews offered a broad analysis of AI models for IVOCT analysis,^29,30^ we specifically focus on the systematic methodological pitfalls in the current literature. We assess the risk of bias (ROB) in the literature, incorporating a quality screening stage to ensure that only papers with sufficiently documented methodologies are reviewed. We also provide detailed recommendations across four domains: (i) considerations for collating IVOCT imaging datasets intended for public release; (ii) methodological considerations for AI/ML researchers and specific issues regarding validation of results; (iii) specific issues regarding the reproducibility of results; and (iv) considerations for reviewers conducting peer reviews of manuscripts.

## Methods

### Review strategy and selection criteria

#### Initial screening

To obtain the screening population, we searched Scopus for all whose titles or abstracts contained either ‘OCT’ or ‘optical coherence tomography’ along with either ‘deep learning’, ‘artificial intelligence’, ‘AI’, ‘machine learning’, ‘neural networks’, ‘auto’, ‘ML’, or ‘net’). This search was not case sensitive and returned 8600 papers whose titles and abstracts were pre-screened to exclude those papers focussed on retinal IVOCT imaging and retain those focussed on coronary arteries (detailed search criteria are detailed in the Supplementary Information).

#### Title and abstract screening

This was performed by three independent reviewers with conflicts resolved via consensus.

#### Full-text screening

Two reviewers performed the full-text screening with conflicts resolved by consensus with a third reviewer.

#### Quality screening

This stage aimed to exclude those DL papers, which had poor quality documentation of critical details necessary for reproducibility of the method described. We followed the approach in Roberts *et al*.^28^ comparing manuscripts with the Checklist for Artificial Intelligence in Medical Imaging (CLAIM)^31^ and excluding those papers, which failed any of eight mandatory criteria (detailed in the Supplementary Information). The full CLAIM checklist is reported for each paper, which passes the quality screen. Traditional ML papers are assessed using the radiomic quality score (RQS)^32^ guidelines but no papers were excluded on the basis of their score.

#### Assessing the risk of bias in studies

In order to assess bias in the datasets, predictors, outcomes, and model analysis in each paper, we use the Prediction model Risk Of Bias ASsessment Tool (PROBAST) of Wolff and coworkers.^33^ Papers that passed the quality screening stage were split among two reviewers to complete the PROBAST review, with conflicts resolved by a third reviewer.

#### Data extraction

Four reviewers extracted data from the manuscripts, with two reviewers considering each manuscript and resolving conflicts. The full dataset is in the Supplementary Data and forms the basis of this review.

## Results

### Study selection

**Figure 2:**
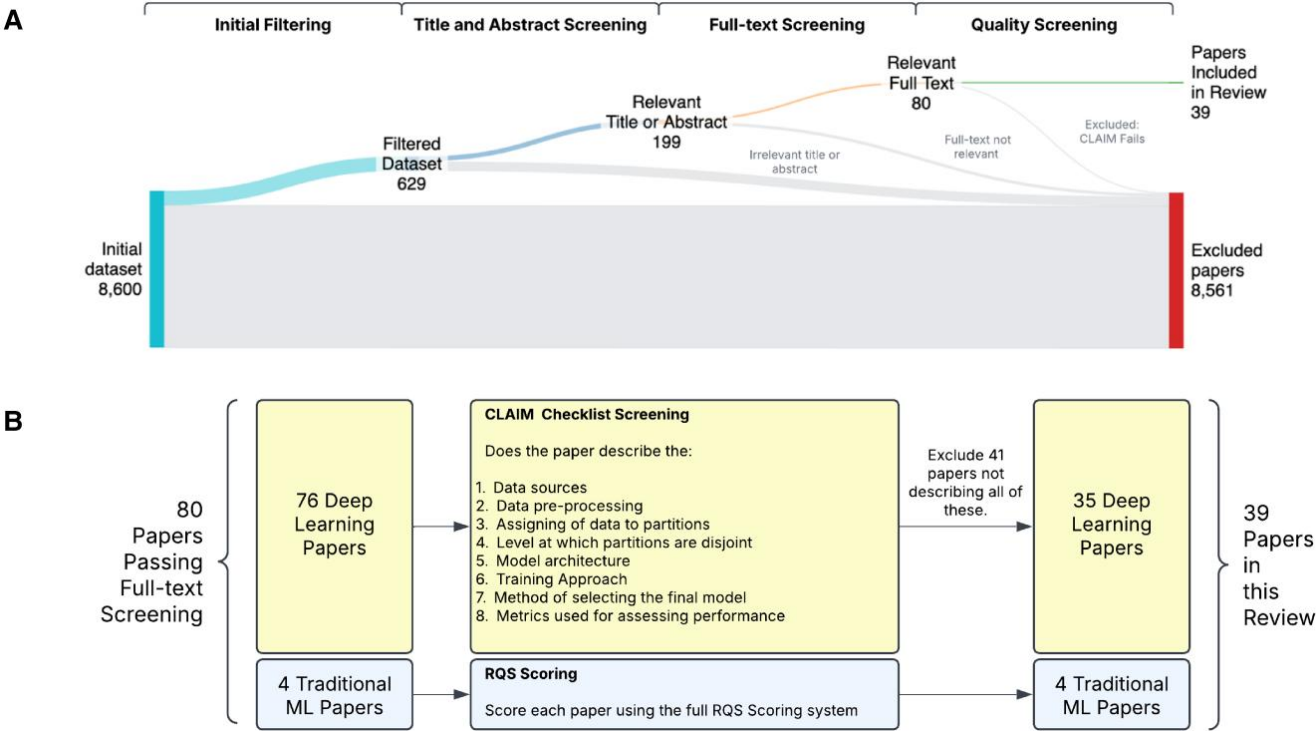
(Top) PRISMA diagram indicating the inclusion/exclusion of papers at each stage of the review. (Bottom) Detail of the quality screening stage.

We identified 8600 papers that satisfied our search criteria and 199 had abstracts or titles relevant to this review, i.e. developing ML methods using IVOCT imaging for diagnostic modelling of CAD. After full-text screening, 80 papers remained and quality screening retained 39/80 papers for consideration in this review (*Table 1*). Of these, 33/39 were pure DL papers and 4/39 we refer to as traditional ML papers (i.e. non-DL ML papers). Two papers developed a hybrid of both approaches^42,68^ (*Figure 2*).

**Table 1:** Key data extracted for each paper

Authors	Model type	Data source	Data format	Split level	Patients	PROBAST	
						Participants	Analysis
Araki *et al*.^34^	Obj. Detection	JP, USA, CN, BE	RGB	Patient	581	Unclear	High
Avital *et al*.^35^	Segmentation	IL	RGB	Frame	Unclear	Low	Low
Cheimariotis *et al*.^36^	Classification	GR, JP	RGB	Patient	33	High	Low
Chu *et al*.^37^	Segmentation	AUS, USA, JP, ES, CN	RGB	Pullback	391	Low	Low
Gharaibeh *et al*.^38^	Segmentation	USA	Raw	Lesion	Unclear	Unclear	High
He *et al*.^39^	Obj. Detection	CN	Raw	Patient	18	Low	Low
Holmberg *et al*.^40^	Segmentation	GER	RGB	Patient	51	High	Low
Huang *et al*.^41^	Segmentation	CN	RGB	Patient	70	Unclear	Low
Kolluru *et al*.^42^	Classification	USA	Raw	Lesion	Unclear	Unclear	Low
Kolluru *et al*.^43^	Segmentation	Unclear	Raw	Patient	48	High	Low
Lee *et al*.^44^	Classification	USA	Raw	Lesion	79	Low	Low
Lee *et al*.^45^	Classification	USA	Raw	Lesion	49	Low	Low
Lee *et al*.^46^	Segmentation	IT	Raw	Hybrid	68	High	Low
Lee *et al*.^47^	Segmentation	USA	Raw	Unclear	Unclear	High	Low
Lee *et al*.^48^	Segmentation	USA	Raw	Lesion	55	High	High
Lee *et al*.^49^	Segmentation	USA	Raw	Lesion	41	High	Low
Lee *et al*.^50^	Segmentation	USA	Raw	Frame	41	High	High
Lee *et al*.^51^	Segmentation	IT, USA	Raw	Pullback	151	Unclear	Low
Li *et al*.^52^	Segmentation	CN	Raw	Patient	45	Unclear	Low
Liu *et al*.^53^	Classification	CN	Grayscale	Patient	60	Unclear	Low
Liu *et al*.^54^	Obj. Detection	CN	Raw	Frame	Unclear	High	Low
Liu *et al*.^55^	Segmentation	Unclear	RGB	Hybrid	540	High	Low
Liu *et al*.^56^	Segmentation	USA	RGB	Pullback	Unclear	High	Low
Min *et al*.^57^	Obj. Detection	KR	Raw	Patient	667	Unclear	High
Niioka *et al*.^58^	Obj. Detection	JP	Raw	Patient	1791	Unclear	Low
Oliveira *et al*.^59^	Segmentation	BR	Grayscale	Patient	51	Unclear	Low
Park *et al*.^60^	Classification	JP, USA, CN, BE	RGB	Patient	581	Unclear	Low
Prabhu *et al*.^61^	Classification	USA	Raw	Patient	49	High	High
Rajkumar *et al*.^62^	Segmentation	MY	RGB	Frame	Unclear	High	High
Ren *et al*.^63^	Segmentation	JP	RGB	Lesion	3	High	High
Roy *et al*.^64^	Obj. Detection	USA	Raw	Lesion	Unclear	Unclear	Low
Shalev *et al*.^65^	Segmentation	USA	Raw	Lesion	Unclear	Unclear	Low
Shalev *et al*.^66^	Classification	USA	RGB	Patient	287	High	High
Shi *et al*.^67^	Obj. Detection	CN	Grayscale	Lesion	Unclear	High	Unclear
Sun *et al*.^68^	Obj. Detection	CN	Grayscale	Patient	83	Unclear	Low
Sun *et al*.^69^	Obj. Detection	CN	Raw	Frame	Unclear	High	Unclear
Sun *et al*.^70^	Obj. Detection	CN	RGB	Frame	Unclear	Unclear	Low
Wu *et al*.^71^	Segmentation	CN	RGB	Patient	70	High	Low
Yin *et al*.^72^	Classification	CN	RGB	Lesion	31	High	High

Obj, Object; AUS, Australia; BE, Belgium; BR, Brazil; CN, China; ES, Spain; GER, Germany; GR, Greece; IL, Israel; IT, Italy; JP, Japan; KR, South Korea; MY, Malaysia; USA, United States of America.

#### Paper quality screen

Quality screening was performed on 80 papers. Of the 76 DL papers screened using the CLAIM checklist,^31^ over half (41/76) were excluded for failing mandatory CLAIM checklist criteria (detailed in the Supplementary Information). 21/41 failed just one, 17/41 failed two, and 3/41 failed three or more. The two most common reasons for failing the quality screen were insufficient documentation of the data source (31/76) and description of the training approach (13/76).

In the next stage, DL papers were evaluated using the full CLAIM checklist, whilst traditional ML papers were assessed with the RQS^32^ checklist to establish how adherent the manuscripts were to established guidelines for manuscript completeness.

##### DL paper quality screen

Only 4/35 papers failed more than 10 items on the 42-point CLAIM checklist and 29/35 failed more than five. The five most common items not satisfied were missing: robustness/sensitivity analysis of models (34/35), details for manual annotation tools (28/35), external model validation (28/35), patient demographics (28/35), and clear details on inclusion/exclusion to obtain patient populations (25/35).

##### Traditional ML paper quality screen

The four papers assessed using RQS received scores of 4,^62^ 5,^65^ 6,^66^ and 8.^61^ No papers reported calibration statistics, statistical significance for discrimination statistics, the potential clinical utility, cost-effectiveness nor shared code, data or models. Only two papers performed feature reduction when selecting features.^61,66^

### Datasets

#### Sources

**Figure 3:**
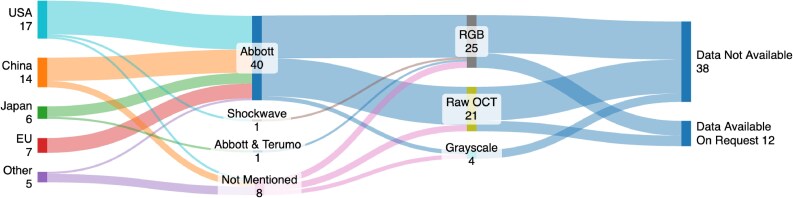
Overview of data sources and modalities in the literature.

To train generalizable and reproducible ML models, it is best practice to train using data from multiple sources, representative of the patient populations under study, and ideally available for other practitioners to access and train/validate against. However, the majority of papers (35/39) used entirely private data sources (one used both a public and private dataset^55^) and 32/39 used a single data source. Data were primarily from the USA (17/39) and China (14/39) with only 5/39 papers^34,36,37,51,60^ using data from multiple countries (*Figure 3*). Only one public dataset was identified but was not available at the time of review. One paper^41^ stated that their data would be released but it had not at the time of this review.

#### Acquisition

To ensure wider generalizability of ML models, the datasets should be acquired from multiple catheter manufacturers, and the input images processed in the same format. However, only 31/39 papers detailed the catheter used to acquire their IVOCT images, 30/31 used Abbott manufactured catheters (or prior named corporate entities) for model training and the other used a Shockwave catheter.^56^ In one paper,^37^ images acquired using a Terumo catheter are used in the external validation cohort and another paper,^55^ which relies on two datasets, used Abbott catheters for one dataset but did not disclose the manufacturer for the other. In 20/39 papers, the raw catheter output was used to train the models, with 15/39 using an artificially coloured RGB image exported from the Abbott offline OCT review software (in a golden colourmap) and 4/39 using grayscale images.

#### Ground-truth annotation

Accurate annotation of ground truth in IVOCT images, often by trained experts using specific software, is critical for high-quality model training and subsequent validation. However, 8/39 papers^36,53,56,61,62,64,67,69^ did not detail who performed manual annotation of ground-truth data, while 9/39 reported one annotator was involved,^34,39,44,59,63,65,66,68,70^ 17/39 reported two and 5/39 reported using more than three annotators.^37,41,54,58,72^ Only 13/39 papers provided details for the specific annotation tools used, namely Abbott's offline review software,^34^ MATLAB's Video Labelling Tool,^62^ Amira,^52,68^ ImageJ,^59^ OCTOPUS,^44,49–51^ LabelMe,^40,63^ Pair,^41^ and custom in-house software.^66^

#### Cohort selection

AI is being proposed for identification of native atherosclerosis, but also for stent optimization and complications of PCI, and ideally any model should be generalizable across different types of CAD and vessels. However, only 19/39 papers reported their study inclusion criteria and only 15/39 papers specified exclusion criteria. One paper disclosed exclusion criteria for one of their datasets but not the other.^55^

#### Sample sizes

Coronary IVOCT pullbacks may be obtained from the same or different arteries from the same patient and on multiple occasions. However, datasets for AI models should comprise large numbers of independent pullbacks, and generalizability/reproducibility claims should not be made based on performance on highly selected IVOCT frames, for example those with ‘classical’ architecture, known measurements, and an absence of imaging artefacts, which may not be reflective of real-world clinical practice. Manuscripts which split at the patient, pullback, lesion, and frame level considered a median of 69, 271, 52, and 41 patients, respectively although the number of patients is not mentioned in 1/3 models that split at pullback-level, 6/14 that split at lesion-level, and 6/7 that split at frame-level. Only 8/39^34,37,55,57,58,66^ papers had a dataset consisting of more than 100 patients. Surprisingly, 33/39 papers used only selected frames from pullbacks in their model development, rather than the whole pullback, with no documentation for how these subsets of frames were selected and real-world applicability is consequently limited.

### Model development

#### Outcomes of interest

**Figure 4:**
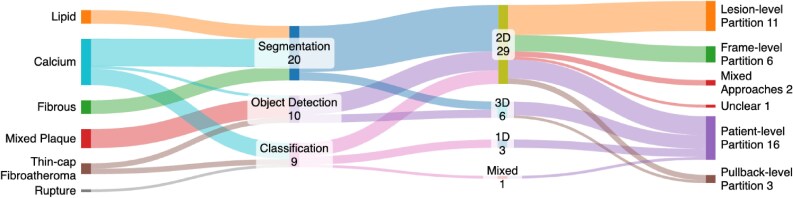
Overview of model development strategies in the literature.

The power of AI to identify and measure tissue types on IVOCT, and thus classify plaques, depends upon accurate localization of tissues with a range of different appearances, using dense segmentations, bounding boxes or A-line- or patch-based classification (*Figure 1*). 20/39 papers described segmentation models for calcium (14/20), lipid (5/20), or fibrous (5/20) plaques, and 10/39 papers described methods which identified a bounding box around tissues including calcified (1/10) or mixed plaques (7/10) and thin fibrous caps (2/10). Classification models were developed in 9/39 papers, focussed on classifying entire frames as showing lipid and calcium plaques (7/8), rupture^60^ (1/8), and thin cap fibroatheromas (1/8).^55^ Three of these papers describe classification models operating on the A-line raw IVOCT data and aimed to distinguish lipid vs. calcium.^36,43,61^ (*Figure 4*).

#### Dataset partitioning

Partitioning datasets is required to derive training, validation, and holdout/test cohorts for model development and evaluation. Interestingly, only 16/39 papers partitioned their datasets at level of the patient, whereas 3/39 partitioned^37^ at pullback-level, 11/39 at the lesion-level, and 7/39 at the frame-level. One paper^46^ trained both a classifier and segmentation models, using a patient-level split and lesion-level split respectively. One paper^55^ trained a model independently for two datasets, with a lesion-level split for one and an unclear approach for the other. Finally, one paper was unclear in how the data had been split.^47^ Failing to split data at the patient-level risks data from the same patient, pullback or lesions being used for both training and model evaluation and the likelihood of optimistic performance reporting. Most papers (21/39) used cross-validation to develop their models, with 9/39 using a fixed internal validation set and 7/39 relying only on internal holdout data. One paper^63^ was unclear in how the model was evaluated and another^55^ developed two models, one using cross-validation and another using a fixed internal validation cohort.

#### Data pre-processing

Despite raw IVOCT imaging data being acquired in polar co-ordinates, most papers (18/39) performed their analysis on Cartesian transformed images, which are more familiar to clinicians, and two used both polar and Cartesian images^52,55^ (*Figure 1*). Real-world IVOCT pullbacks contain multiple artefacts,^8^ a significant proportion of which affect interpretation of the underlying vessel wall. However, only 14/39 papers applied denoising techniques to suppress these artefacts (e.g. using Gaussian and Gabor filters). Segmentation quality and measurements can be greatly affected by image resolution and size, both of which are commonly standardized during pre-processing. The resampled image size is mentioned in only 24/39 papers with 5/24 papers considering a Cartesian image resolution above 512 × 512 pixels^36,37,41,53,56^ and 13/24 using polar images of resolution above 400 × 900 pixels.

#### Model inputs

IVOCT images are acquired using a spinning catheter sampling radially as it is pulled along the artery; these radial samples are stacked to form the 2D frames of the final image. The 2D frames themselves are stacked to form a 3D volume. A majority of papers (29/39) developed their models using 2D frames as inputs with 6/39 considering the pullback as 3D volumetric data^34,37,39,52,68,71^ and one used both 2D and 3D imaging.^60^ 3/39^36,43,61^ papers focussed on the 1D A-line data and none of the papers consider the spiral nature of IVOCT data acquisition in their modelling approach.

#### Model architecture

There is a rapidly increasing selection of available AI applications and model architectures reflected in the published literature. Of the 20 papers describing segmentation models, 16/20 built upon existing DL architectures including: U-Net-like (5/16),^35,37,40,42,52^ SegNet (4/16),^38,48,59,63^ Deeplabv3+ (4/16),^44,48–50^ Transformer-based models (2/16),^53,56^ and SegResNet^51^ while the other DL-based papers trained using custom architectures. The 2/20 papers which focussed on traditional ML algorithms evaluated a random forest,^62^ XGBoost,^62^ and support vector machines^65^ for pixel-level classification. For the 10/39 papers describing object detection applications, all used DL with 2/10 papers using a Faster R-CNN^69,70^ and ResNet,^39,67^ whilst the rest employed a highly diverse range of architectures including a vision transformer,^34^ DenseNet,^57^ Inception-V3,^58^ EfficientNet,^58^ Mask RCNN,^68^ VGG,^54^ and autoencoders.^64^ For the nine classification models, 3/9 papers described DL models established architectures,^36,60,72^ 4/9 used custom architectures^43,45,47,55^ and two used a support vector machine and random forest for A-line classification^61^ and plaque classification.^66^

#### Metrics

In addition to different model architectures, IVOCT segmentation performance was described using several different metrics. The Dice score was most frequently used (14/20) to assess IVOCT segmentation model performance, whilst pixel-based accuracy/sensitivity, Intersection Over Union or F1-score were used in other papers. Classification model performance was assessed using a diverse range of metrics, namely recall/sensitivity, precision, specificity, accuracy, F1 score, negative predictive value, the area under the receiver operating characteristic curve, and Bland–Altmann agreement. Object detection models used many of the classification metrics and the overlap rate, mean average precision and Brier score.

### Risk of bias assessment

AI models can be very prone to bias, most commonly arising from the participants, predictors, outcome, and analysis (see Supplementary Data). The ROB and concerns of applicability were assessed for all papers using the PROBAST guidance. We found a high ROB in at least one of the four domains in 22/39 papers (see Supplementary Data).

#### Participants

The ROB was rated as low for the participants domain in only 5/39 papers,^34,37,57,58,60^ with a high ROB found in 19/39 papers (see *Table 1*). This was primarily due to the selection of samples at region-, segment- or frame-level, with a risk of validating the model using patients that the model was trained upon. Risks of bias were also increased due to: selecting a subset of pullback frames without clear inclusion/exclusion criteria; using only well-defined samples and dropping those with equivocal findings; only sampling from selected plaque pathologies; using a data subset where the demographics varied significantly from the full cohort; and only using pullbacks from patient groups where particular plaque compositions were significantly enriched. The ROB was rated as unclear for the Participants domain in 15/39 papers in which insufficient demographics and recruitment information was given, and was found for both private and public datasets.

#### Predictors

Almost all papers (35/39) developed DL models in which the predictors are learned by the model. There can be a very large number of predictors in DL models, weighted by learned parameters. Therefore, it is unknown what the predictors are, their number or what they represent and, consistent with Roberts *et al*.,^28^ the ROB was rated as unclear for these papers. The remaining four papers^61,62,65,66^ were found to have a low ROB, relying on pre-defined hand-engineered features derived from the images.

#### Outcome

All papers included in this review described models for classifying plaque types or localizing their components with these outcomes defined using consensus recommendations.^4^ Therefore, all papers were rated low ROB in this domain.

#### Analysis

Most papers (27/39) were found to have a low ROB for their analysis (see *Table 1*). High ROB was found in 10/39 papers due to small sample sizes or inappropriate performance evaluation.^70^ Two papers had an unclear ROB^38,39^ as they did not report the proportion of positive samples in their dataset.

### Code and model availability

AI model performance can vary greatly between centres and datasets, and external reproduction of results is required to be confident of their generalizability. However, no papers provided detailed instructions or open-source code to allow for the external reproduction of results. Most papers also did not share any data (30/39), with one stating that it will be released after publication^41^ (but is unavailable at the time of this review) and the remainder stating that data were available on reasonable request. Many papers did not mention the software in which their models were implemented (15/39) and of those which did, most used MATLAB (13/39) or Python (11/39). One paper used a commercial tool OctPlus.^37^

## Discussion

IVOCT is the highest-resolution modality for the imaging of the coronary arteries, that is widely available, and the only one able to identify and measure high-risk thin fibrous caps. In 2024, European guidelines were updated to strongly recommend the use of IVOCT to guide stenting of complex lesions. However, accurate interpretation of IVOCT imaging requires significant training and is a barrier to the scaling of IVOCT adoption. AI models hold significant promise for far faster and more scalable analysis compared with a human reader. Therefore, with increasing availability of IVOCT data and the ability to analyse them with DL tools and hardware, it is only natural that we see increasing appetite from researchers to develop AI models for interpreting IVOCT images. However, we found that although published studies show considerable promise and potential in this field, many are burdened with methodological and reporting deficiencies, with most of the reviewed literature not ready for clinical application. We have identified issues around dataset documentation, methodologies, reproducibility, and biases in study design, which we now summarize and suggest recommendations to improve the evidence base to allow for wider adoption of AI tools for automated IVOCT interpretation.

In general, there is a strong preference in the literature towards training of DL models rather than more traditional radiomics approaches, with only three focussing on the latter (only one since 2020). Additionally, very few papers developed models to give a frame-level classification for disease status, with all others focussed on localizing the disease through segmentation and object detection within the image. All the papers which disclosed their catheter manufacturer reported using images acquired by Abbott catheters for model training, likely due to data availability with Abbott distributing the majority of IVOCT catheters globally. Consequently, the majority of models are likely applicable only to Abbott acquired imaging.

### Image acquisition and datasets

#### Image acquisition

IVOCT images are acquired by a spinning catheter pulled down an artery sampling 1D radially (A-lines). After each 360° rotation, the 1D signals are stacked to give a 2D radial grayscale image. These images are then often exported from the Abbott offline review software which converts images to Cartesian an artificial ‘golden’ colourmap that is most familiar to clinicians. These 2D images are then stacked into pseudo-3D images. No papers in this review considered the spiral nature of the acquisition in their modelling, rather focussing the IVOCT images as 2D/3D acquisitions. It is unknown whether incorporating the acquisition technique into the methodology will improve model quality by reducing known artefacts (such as seam artefacts) that occur due to stacking into 2D frames.

#### Dataset sizes

We found that many papers used relatively small IVOCT datasets for model development with a median of 55 patients and only six studies using datasets with more than 100 patients. Developing models using small-scale datasets risks introducing potential biases into the model, limiting its generalizability, and findings should be approached cautiously. For DL models, it is common to require many thousands of training samples due to their over parametrization, which is more achievable at the 2D frame level but often hard to achieve (and of unclear necessity) at the 3D pullback-level.

#### Dataset diversity

The acquisition quality for IVOCT images is heavily influenced by the expertize of the interventional cardiologist performing the procedure. Therefore, a diversity in image quality can be achieved by collecting images from procedures performed by clinicians of varied experience. AI models developed using this data would then have wider applicability to images of heterogeneous quality.

Moreover, the real-world diversity of IVOCT imaging data collected from patients of different backgrounds, it is unfortunate that only six studies utilized data from multiple countries with others training primarily with data from the USA and China. This bias in geographic scope will likely significantly limit model applicability, and it is of primary importance to ensure a diversity of patients and of disease profiles to avoid bias in the model and enhance its applicability.

Finally, it is also important to note that many datasets have been curated by academic and clinical groups who are likely trained in a similar way and follow similar labelling protocols. As inter-reader variability of the annotations can be significant, we would encourage that there is a diversity and independence of the data annotators used for model development and evaluation. Inter- and intra-reader variability for annotation quality should also be reported.

#### Ground truth

Clinicians are trained to interpret the artificially coloured Cartesian IVOCT images, derived from the raw grayscale polar IVOCT data using the Abbott golden colourmap. Surprisingly, however, in around half of papers, it is the raw image data that is used to train models and it is generally unclear how the ground-truth segmentation labels were generated when these data are never observed in clinical practice. We may presume that experts label the images using the coloured IVOCT and the annotations are propagated to the final polar domain, but this is not mentioned.

For classification models, if the disease labels (e.g. adaptive/pathological intimal thickening and thin cap fibroatheromas) themselves are assigned based on the IVOCT images it risks introducing ‘incorporation bias’ as the classification labels are not independent of the images. To break this link, authors should evaluate model performance with labels assigned independently, e.g. from histopathology slices.

#### Inclusion/exclusion criteria

Reporting inclusion and exclusion criteria is crucial for understanding a model's training population and likely limitations of applicability. Their absence was particularly striking in our review, with only 15 papers providing detailed inclusion criteria and exclusion criteria reported in only 12 papers. The inclusion and exclusion criteria varied widely between studies (depending on area of focus), highlighting the heterogeneity in patient populations used for training and underscores the need for transparent reporting to allow for fair study comparisons and improve reproducibility.

### Methodologies for model building

#### Dataset partitioning

Most papers did not partition their datasets at the patient level and used only an undisclosed subset of frames from the pullback for training and evaluation. This leads to a high-risk of data leakage in the literature whereby frames from the same patient are being allocated to train/validation/holdout cohorts, and similar plaque features from adjacent frames may inadvertently influence model performance and lead to optimistic performance.

#### Cartesian vs. polar

Most papers used Cartesian transformed images in their models whereas the image signals are acquired in the polar domain. Transforming the signals from the polar to Cartesian domain is not reversible and necessarily introduces data loss, as pixels are squeezed together near the catheter and also artefacts are potentially introduced as the intensity values are stretched across several pixels further from the catheter. Ground-truth labels can be transformed between these domains, allowing for experts to label the Cartesian images in the form they are most used to.

#### Validation issues

For model development, twice the number of papers employed cross-validation against those using a single fixed validation cohort to evaluate their model performance. Most models developed using a single private dataset and external validation is absent in most papers, both of which raise important concerns around the generalizability of the models to new populations. Furthermore, with the widespread lack of disclosure of inclusion/exclusion criteria and demographic information on the training population, our review of bias assessment gives a high concern in the ability of the models to generalize to new populations for a majority of papers.

#### Deep learning vs. traditional machine learning

There were only three traditional ML papers included in this review from 2016, 2019, to 2021 reflecting the more recent adoption of DL methods for AI-based image analysis. These all relied on traditional computer vision and radiomics techniques, such as applying Gabor, Gaussian, and Laplacian of Gaussian filtering to images, extracting pre-defined features and then fitting of classification models and clustering techniques. These pre-defined features have the benefit of providing explainable predictors, compared with DL models, which must be analysed after training with e.g. attention maps using GradCAM.^73^

### Reproducibility of the existing literature

#### Manuscript documentation

In general, the literature is poorly documented for reproducibility, as shown by the quality screening, which removed over half of all papers considered. This was primarily due to insufficient documentation of data sources and model training descriptions. However, as discussed previously, the provenance of data, the inclusion/exclusion criteria and the demographics of the training cohort are critical for accurately understanding the cohort that the results may reproduce. In addition, for those models developed and assessed against selected frames from pullbacks, rather than full images themselves, it is hard to reproduce this selection without a sufficient description of the process. Similarly, without sufficient descriptions of the model training procedure, it is impossible to reproduce the results of any experiments.

#### Open science

The only public dataset that is considered in the literature, CCCV-IVOCT from 2017, appears no longer to be available or accessible. This is very unfortunate as it prevents the transparent benchmarking of model performance and is in contrast with many application areas of AI. We also encourage authors to consider the FAIR^74^ (findability, accessibility, interoperability, and reuse) principles for scientific data management when releasing datasets publicly to ensure they can be readily used by other researchers. Clearly, there are logistical, cost, ownership and ethical concerns with releasing open-sourced datasets, but it has been highly successful in other clinical domains and enabled a significant increase in AI model development. For example, there are several large (curated) datasets publicly available for AI model development using Chest X-Rays,^75,76^ each being cited thousands of times.

Similarly, for AI methods in other clinical domains it is common for academic papers to openly share code and trained models to allow for an easier assessment of both whether the proposed model outperforms prior models and how well models generalize to new data. Without such convenience, each author must develop and validate their tools in isolation without performance benchmarks to compare against. However, we neither find that no papers share their code nor were trained models shared.

### Recommendations

In *Table 2* we summarize the recommended actions that are taken by researchers, authors and journals to improve the quality of the models being developed. Complementing these, Föllmer *et al*.^30^ have provided a detailed roadmap describing more broadly how AI methods should be developed for the imaging of atherosclerotic plaque in coronary arteries.

**Table 2:** A summary of the recommendations identified in this review

Validation strategies: Before developing models, developers must have a clear validation strategy with external datasets held out during model development for assessing the generalizability of the models.
More open science: The IVOCT research community should aim to publicly release (and permanently archive) more IVOCT datasets from different demographics, to allow for better (transparent) benchmarking of model performances which comply with FAIR principles. The research community should explore incentives, policy changes or establishing international consortia and collaborations, providing potential routes for the collection of large, diverse, standardized, and openly available datasets. Progress by the research community would be accelerated by a more transparent and open sharing of code and models to allow for easier assessment of how well models generalize to new data. Platforms such as Hugging Face and Code Ocean allow for rapid deployment of models reproducibly to a wide audience of model developers.
Encourage full pullbacks use: Models should be trained and evaluated against full IVOCT pullbacks. If full pullbacks are not used, it should be clearly stated as a limitation of the study.
Dataset partitioning: Dataset sizes should be primarily reported at the patient level and partitioning into training, internal validation, and holdout cohorts should be performed at that level.
Dataset sourcing: Standardized reporting guidelines should be adopted to ensure transparency regarding dataset origins and patient demographics. Authors should adhere to established guidelines for reporting inclusion and exclusion criteria to ensure transparency and consistency in reporting. Model developers should avoid building models using collections of 2D images, which are of unknown provenance, as the inherent structural relationships between images from the same pullback give rise to risks of reporting overly optimistic performance.
Dataset diversity: Datasets should be diverse with respect to (a) the expertize of the interventional cardiologist obtaining the images, (b) patient demographics, (c) patient disease state, and (d) the expertize and independence of the experts that are annotating the data used for training. The community should design incentives or policies to promote.
Ground truth: Manuscripts should clearly disclose how the images were assigned their ground-truth labels. For segmentation models this should include information on whether the artificially coloured RGB images were those labelled and the software used. The known performance differences between experienced and junior IVOCT readers^11^ means that it is important to disclose the annotator's experience level. For classification models, the model should be evaluated using data whose labels have been derived independent from the IVOCT images themselves.
Improving documentation: During manuscript drafting, authors should assess their papers against established standards such as CLAIM,^31^ RQS,^32^ PROBAST,^33^ REFORMS (Reporting Standards for Machine Learning Based Science),^77^ TRIPOD (Transparent Reporting of a Multivariable Prediction Model for Individual Prognosis or Diagnosis),^78^ and QUADAS (Quality Assessment of Diagnostic Accuracy Studies).^79^ For manuscript reviewers and journal editors, we recommend using these checklists to identify weaknesses in methodology reporting when giving feedback on manuscripts.

### Limitations of our review

In this review, we have focussed purely on the methodological aspects of IVOCT AI model development; however, we have not considered the associated concerns around ethics, regulatory considerations, the challenges of clinical deployment and how the biases we have identified may impact the final utility. Additionally, we have not fully explored the root causes for many of the systemic issues identified, including the lack of open-source datasets, nor have we considered fully the impact that open-source data, code and models would have on the development of AI tools for IVOCT. We have only examined the English language literature. We have not considered commercially available AI-based tools for IVOCT interpretation, such as Ultreon (Abbott Laboratories, USA), AutoOCT (Octiocor Ltd, UK), OCTPlus (Pulse Medical Imaging Technology, China), and DeepOCT (Spectrawave), where the technology is proprietary, as the focus of our review is on the methodology for emerging technologies.

In our PROBAST review of the Predictor domain, we assigned the DL-based papers a ROB of Unclear. To appropriately assess the ROB, PROBAST requires us first to ‘list and describe predictors included in the final model’ and then answer three questions: Were predictors defined and assessed in a similar way for all participants? Were predictor assessments made without knowledge of outcome data? Are all predictors available at the time the model is intended to be used? For DL-based papers, it is not possible to list or describe the predictors used by the model as the influence of imaging features on the model predictions is unclear due to learned model parameters. Additionally, we cannot know whether the predictors used are the same for all participants nor whether shortcuts have been learned between irrelevant image features and outcomes.^80^ We also acknowledge that in the RQS screening we do not assign a minimum score for excluding papers from consideration (as there are so few to consider), however for DL papers, the quality screening stage removes many papers from consideration. Successfully passing through the quality screening does not suggest an equivalence between the CLAIM and RQS assessed papers and they cannot be compared. Similarly, failing the CLAIM screening does not reflect on the quality of the paper, or minimize its contribution, but only compares against the mandatory criteria.

## Conclusions

IVOCT use is demonstrated to save lives and reduce heart attacks^24,25^ in a range of clinical scenarios. However, its utilization remains low. AI-based tools hold the promise to allow for rapid and automated interpretation of IVOCT images to aid clinical decisions. We believe it is incredibly important that AI-based tools have the highest possibility of clinical translation and we have examined the published literature on AI/ML methodologies applied to the diagnosis of CAD using IVOCT, focusing on the quality, reproducibility and potential clinical utility of these methodologies. To enhance the likelihood of these models being integrated into future clinical trials, we present detailed recommendations, including (i) using datasets with precise and transparent descriptions of data collection, pre-processing, and any transformations applied; (ii) using datasets from outside of the USA and China to assess generalizability to more geographically diverse populations; (iii) providing thoroughly documented manuscripts with detailed methodologies to ensure that studies can be accurately replicated; (iv) conducting comprehensive external validation with independent datasets to ensure the model's performance is reliable and generalizable across different populations and clinical settings.

## Supplementary Material

ztaf053_Supplementary_Data

## Data Availability

The data underlying this article are available in the article and in its online supplementary material.
