# Supplementary material for: Review and recommendations for using artificial intelligence in intracoronary optical coherence tomography analysis
Source: Eur Heart J Digit Health. 2025 May 15;6(4):529–39. doi: 10.1093/ehjdh/ztaf053 (PMC12282360; doi:10.1093/ehjdh/ztaf053)
Supplement: ztaf053_Supplementary_Data [file ztaf053_supplementary_data.zip › Supplementary_Information.docx]

**Supplementary Information**

*Search Strategy*

To obtain the screening population, we searched SCOPUS for all whose titles or abstracts contained either “OCT” or “optical coherence tomography” along with either “deep learning”, “artificial intelligence”, “AI”, “machine learning”, “neural networks”, “auto”, “ML” or “net”). This search was not case sensitive and returned 8,600 papers whose titles and abstracts were pre-screened to require one of the terms "coronary", "arteries", "artery", "atherosclerosis", “plaque", "atherosclerotic", "myocardial infarction", "angina", “coronary bypass surgery", "coronary angioplasty", "stent", "thrombosis", "catheter", "carotid", "calcium", "lipid", "fibrous", tcfa" and "lumen". This set of papers contained many retinal IVOCT imaging papers which we excluded by removing those which contained at least one of "retina", "retinal", "macula", "fovea", "optic nerve", "choroid", "ophthalmology", "ophthalmologist", "ocular", "cornea", "lens", "iris", "pupil", "conjunctiva", "sclera", "eye disease", "eye disorder", "vision impairment", "blindness", "glaucoma", "cataract", "diabetic retinopathy", "age-related macular degeneration", "retinitis pigmentosa", "uveitis", "macular edema", "optic neuritis", "conjunctivitis", "strabismus", "amblyopia", "myopia", "hyperopia", "astigmatism", "color blindness", "night blindness", "dry eye syndrome", "floaters", "flashes", "presbyopia" or "ocular hypertension". This retained 629 papers for title and abstract screening.

*Quality screen exclusions*

The mandatory CLAIM criteria required by each paper quoted below copy those of Roberts et al. (https://doi.org/10.1038/s42256-021-00307-0) they are repeated below for completeness. These must be satisfied in any deep learning paper included in the review.

- Data sources [item 7]. The data sources must be clearly identified to allow reproducible collection of the same datasets. If only a subset of particular datasets has been used, then it must be detailed how this subset was acquired and how this could be reproduced.
- Data pre-processing steps [item 9]. The data we consider is primarily imaging data, therefore we require that the paper details the pre-processing steps in sufficient detail to reproduce. This includes details on how the image intensities were manipulated before being input to the networks, we expect details about any rescaling of the image resolution and the method for rescaling, the number of colour channels in the input image.
- How data were assigned to partitions; specify proportions [item 20]. For the training to be reproducible, we expect not only the proportions (or number) of images included within each of the training, validation and holdout cohorts but also the number of images with the outcome.
- Level at which partitions are disjoint (e.g. image, study, patient, institution) [item 21]. If a paper has only one image for each patient all obtained from the same center, then we can safely assume these are disjoint at patient level. In the instance that it is clear that there are multiple images for some (or all) patients in the dataset, we expect detail to be given for how the authors mitigated against images appearing in the different partitions for the same patients.
- Detailed description of model, including inputs, outputs, all intermediate layers and connections [item 22]. The construction of the architecture must be reproducible to allow the training to be replicated. Therefore, if a paper uses a common architecture and cites it elsewhere, we then deem this to be satisfied so long as the output layers are all detailed. If a custom architecture is employed, then we expect to be able to reproduce this from the detail in the paper.
- Details of training approach, including data augmentation, hyperparameters, number of models trained [item 25]. The method for training the model must be discussed in enough detail to allow reproduction, this includes details such as the loss function used, the optimizer, the initial learning rate (and any decay used) along with utilization of data augmentation.
- Method of selecting the final model [item 26]. If the authors consider a model that is not trained for a fixed number of epochs, we require that the authors detail how the final model was selected. This could be, for example, use of early stopping criteria to stop training after the validation loss, accuracy or AUC stops improving.
- Metrics of model performance [item 28]. The metrics used to assess the model performance must be commonly used metrics or defined clearly within the paper.

**PROBAST Results**

| Title | |
| --- | --- |
| 5416. Coronary calcification identification in optical coherence tomography using convolutional neural networks | |
| Description of Data (source, inclusion/exclusion, setting and dates, etc.) | |
| The data used in this work was taken from 13 patients suffering from CAD. 51 frames with calcific plaques were manually selected for the experiments. Images were acquired using the St. Jude system.  35 frames for training and 16 for testing. Ground truth was manual delineation by a single reader blinded to automatic segmentation. | |
| Domain 1: Participants | |
| Signalling Questions | |
| 1.1 Were appropriate data sources used, e.g. cohort, RCT or nested case-control study data? | Unclear |
| 1.2 Were all inclusions and exclusions of participants appropriate? | High |
| A. Risk of Bias | |
| **Risk of bias with participants selection (low/high/unclear)** | **High** |
| *Rationale of bias rating:*  R1: As a short conference paper there wasn’t enough information about the participants inclusion/exclusion. It was also unclear why only 51 frames were chosen – there will typically be a lot more calcific frames from 13 pullbacks so selection bias may exist.  The study only included pullbacks with confirmed calcification, which may introduce bias as the algorithm tends to always find at least some calcification.  R2: The outcomes (calcific plaque identification) were assessed using manual segmentation by an independent observer. While this provides a ground truth for comparison, the study does not detail the observer’s expertise or the consistency of the segmentation process. The manual segmentation process can introduce inter-observer variability, leading to potential bias in outcome assessment. Additionally, the use of a small number of frames (16) for testing further compounds this issue, as it may not provide a representative assessment of the model's performance. |  |
| B. Applicability | |
| **Concern that the included participants and setting do not match the review question** **(low/high/unclear)** | **High** |
| *Rationale of applicability rating:*  R1: As discussed above the algorithm was trained only on frames with confirmed calcification, which may limit its generalisability to routine images. It was a very small cohort with only 51 frames from 13 pullbacks which limits its applicability to the general population.  R2: The study’s participants are not representative of the broader population of patients with coronary artery disease (CAD) because only patients with confirmed calcification were included. This narrow selection limits the generalisability of the findings to other patient populations. |  |

| Title | |
| --- | --- |
| 2747. Coronary Artery Fibrous Plaque Detection Based on Multi-Scale Convolutional Neural Networks | |
| Description of Data (source, inclusion/exclusion, setting and dates, etc.) | |
| Total 1000 frames collected at Peking Union Medical College Hospital, China with St. Jude imaging system. It was unclear how many pullbacks and patients were involved. Only frames confirmed as fibrous plaques were used. Ground truth of training images was annotated by 3 readers, but it was unclear whether the annotation was generated/reviewed by multiple readers. Did not mention about the annotation of test set either. 85-15 training-testing split.  A bounding-box detection was developed to find the fibrous region but without depth information. | |
| Domain 1: Participants | |
| Signalling Questions | |
| 1.1 Were appropriate data sources used, e.g. cohort, RCT or nested case-control study data? | Unclear |
| 1.2 Were all inclusions and exclusions of participants appropriate? | High |
| A. Risk of Bias | |
| **Risk of bias with participants selection (low/high/unclear)** | **High** |
| *Rationale of bias rating:*  R1: There was no information on the number of patients and their demographics. The study only used frames of fibrous plaque and not negative controls, which may introduce bias.  R2: The study did not provide sufficient information about the inclusion and exclusion criteria for participant selection. It was unclear how the 14 patients were chosen and whether these patients were representative of the broader population. The lack of detailed demographic and clinical characteristics of the selected patients raises concerns about potential selection bias. This limited transparency makes it difficult to assess whether the study population accurately reflects the target population for which the AI model is intended. |  |
| B. Applicability | |
| **Concern that the included participants and setting do not match the review question** **(low/high/unclear)** | **High** |
| *Rationale of applicability rating:*  R1: The lack of patient inclusion/exclusion criteria may limit the generalisability of the work. Model was developed with frames of fibrous plaque only and may not work well on fibroatheroma or fibrocalcific plaques which are more clinically relevant. The bounding boxes had no depth information and could not be used for fibrous cap thickness quantification either. This makes the approach less suitable for diagnostic or prognostic purposes.  R2: The lack of standardization in feature selection and extraction processes limits the model's generalisability and potential for broader clinical application. |  |

| Title | |
| --- | --- |
| 2582. Deformable deep network atherosclerotic coronary plaque recognition of oct imaging | |
| Description of Data (source, inclusion/exclusion, setting and dates, etc.) | |
| 2000 frames (1000 positive samples with vulnerable plaques and 1000 negative) taken from the 2017 CCCV-IVOCT challenge database with an 80-20 training-testing split. The 300-frame test set of CCCV-IVOCT was not used in this study. No patient information was provided in CCCV-IVOCT. | |
| Domain 1: Participants | |
| Signalling Questions | |
| 1.1 Were appropriate data sources used, e.g. cohort, RCT or nested case-control study data? | Unclear |
| 1.2 Were all inclusions and exclusions of participants appropriate? | Unclear |
| A. Risk of Bias | |
| **Risk of bias with participants selection (low/high/unclear)** | **Unclear** |
| *Rationale of bias rating:*  R1 & R2: CCCV-IVOCT dataset without additional validation. The risk of bias due to frame/patient selection cannot be excluded. |  |
| B. Applicability | |
| **Concern that the included participants and setting do not match the review question** **(low/high/unclear)** | **High** |
| *Rationale of applicability rating:*  R1& R2: No patient inclusion/exclusion criteria or demographics were provided in CCCV-IVOCT dataset. There is a high risk in the applicability and generalisability to routine scans. |  |

| Title | |
| --- | --- |
| 2564. Automated classification of coronary plaque calcification in OCT pullbacks with 3D deep neural networks | |
| Description of Data (source, inclusion/exclusion, setting and dates, etc.) | |
| A total of 24 pullbacks from 24 patients imaged using the St. Jude system at Affiliated Drum Tower Hospital, Nanjing, China between Dec 2016 and Dec 2018. Ground-truth was annotated by a single reader, and each frame was dichotomized as either calcified plaque or non-calcified plaque. There was no information on the number of ground-truth positive and negatives.  All images of each pullback were used in this study. 4000 frames from 18 pullbacks used for training, 344 frames from 2 pullbacks for validation and 800 frames from 4 pullbacks for testing. | |
| Domain 1: Participants | |
| Signalling Questions | |
| 1.1 Were appropriate data sources used, e.g. cohort, RCT or nested case-control study data? | Unclear |
| 1.2 Were all inclusions and exclusions of participants appropriate? | High |
| A. Risk of Bias | |
| **Risk of bias with participants selection (low/high/unclear)** | **High** |
| *Rationale of bias rating:*  R1: There was no information on patient inclusion/exclusion criteria and demographics. The actual number of ground-truth positives and negatives were not given in the paper. It was not possible to determine the plaque type (e.g. mixed, fibrocalcific, etc.) or disease severity, and therefore bias may exist in the participant selection.  R2: The study does not provide detailed information on the inclusion and exclusion criteria for the participants. It appears that the dataset was limited to 24 patients from a single hospital, raising concerns about the representativeness and generalisability of the findings. The absence of clear participant selection criteria may introduce selection bias, as the sample might not reflect the broader population affected by coronary artery disease. The study also lacked information about any stratification of participants based on demographic variables, further heightening the risk of bias. |  |
| B. Applicability | |
| **Concern that the included participants and setting do not match the review question** **(low/high/unclear)** | **High** |
| *Rationale of applicability rating:*  R1: As noted, there was no information on the patient information which may limit the applicability. The authors did not describe the number of ground-truth positives and negatives so there is a high risk in data being not representative in general population. Furthermore, the paper worked on a frame-wise dichotomization into calcified and non-calcified plaques but with no quantification of calcium angle, which may limit its use in diagnosis/prognosis.  R2: The applicability of this study is limited by its small and non-diverse dataset. While the methods and models proposed, such as the ResNet-3D, are promising for classification tasks in IVOCT, the lack of diverse data limits the generalisability of the results to broader clinical settings. Without validation on external datasets, it is unclear whether the model would perform well across different populations or imaging conditions. |  |

| Title | |
| --- | --- |
| 5052. Artificial intelligence and optical coherence tomography for the automatic characterisation of human atherosclerotic plaques | |
| Description of Data (source, inclusion/exclusion, setting and dates, etc.) | |
| Model development involved 509 pullbacks of 391 patients from 3 IVOCT studies including 5 international centres. Images were acquired using the Abbott system. Patient inclusion/exclusion criteria and demography was clearly stated. Annotation was done in core lab and reviewed by another expert. A total of 11,673 frames were split into 9-1 for training and internal testing.  External testing involved 3 core labs, each providing 100 frames with delineated regions from 10 patients, summing to 604 tissue regions from 45 lesions. The region was independently labelled by 3 core labs and evaluated by 4 experts. Both Abbott and Terumo system were used. | |
| Domain 1: Participants | |
| Signalling Questions | |
| 1.1 Were appropriate data sources used, e.g. cohort, RCT or nested case-control study data? | Low |
| 1.2 Were all inclusions and exclusions of participants appropriate? | Low |
| A. Risk of Bias | |
| **Risk of bias with participants selection (low/high/unclear)** | **Low** |
| *Rationale of bias rating:*  R1 & R2: Low risk as summarized in the description. There are still potential issues with the external testing set but unlikely to be substantial: (1) only 10 frames from each patient so there might be a selection bias; and (2) the regions were already provided by core labs, which may lead to anchoring bias in the following expert assessment stage. |  |
| B. Applicability | |
| **Concern that the included participants and setting do not match the review question** **(low/high/unclear)** | **Low** |
| *Rationale of applicability rating:*  R1: Multicentre and multinational study with large number of frames and patients. Also provided external validation with expert consensus as ground truth. Tested in both Abbott and Terumo systems.  R2: The model developed in the study shows promise for broad application in IVOCT. However, the use of data from specific centres, with patients having stable lesions, limits the generalisability of the results to other populations. |  |

| Title | |
| --- | --- |
| 2388. Cascade Faster R-CNN Detection for Vulnerable Plaques in OCT Images | |
| Description of Data (source, inclusion/exclusion, setting and dates, etc.) | |
| Used the 2017 CCCV-IVOCT contest dataset. This involved 2000 frames in polar coordinates for training, among which were 1000 positive samples with vulnerable plaque and 1000 negative samples. Potentially, 500 frames were used for testing but is unclear. No patient information was provided in CCCV-IVOCT. | |
| Domain 1: Participants | |
| A. Risk of Bias | |
| 1.1 Were appropriate data sources used, e.g. cohort, RCT or nested case-control study data? | Unclear |
| 1.2 Were all inclusions and exclusions of participants appropriate? | Unclear |
| **Risk of bias with participants selection (low/high/unclear)** | **Unclear** |
| *Rationale of bias rating:*  R1 & R2: CCCV-IVOCT dataset without additional validation. The risk of bias due to frame/patient selection cannot be excluded. |  |
| B. Applicability | |
| **Concern that the included participants and setting do not match the review question** **(low/high/unclear)** | **High** |
| *Rationale of applicability rating:*  R1 & R2: No patient inclusion/exclusion criteria or demographics were provided in CCCV-IVOCT dataset. There is a high risk in the applicability and generalisability to routine scans. |  |

| Title | |
| --- | --- |
| 2223. Coronary Plaque Characterization from Optical Coherence Tomography Imaging with a Two-Pathway Cascade Convolutional Neural Network Architecture | |
| Description of Data (source, inclusion/exclusion, setting and dates, etc.) | |
| The study involved 2000 frames from 31 patients and consequently 2000 ROIs. No information on patient group or demographics was given. Fibrous tissue and lipid tissue comprised 40% of the whole dataset, respectively, and calcified tissue occupied 20%.  The methodology is a bit confusing. A 6-1 ratio of training and independent validation was described in Methods, but the authors also mentioned cross validation in Experiments and Results. Furthermore, the authors mentioned a test set of 30 samples from 10 pullbacks of 3 patients in the Introduction, but it did not appear anywhere beyond Introduction. | |
| Domain 1: Participants | |
| Signalling Questions | |
| 1.1 Were appropriate data sources used, e.g. cohort, RCT or nested case-control study data? | Unclear |
| 1.2 Were all inclusions and exclusions of participants appropriate? | High |
| A. Risk of Bias | |
| **Risk of bias with participants selection (low/high/unclear)** | **High** |
| *Rationale of bias rating:*  R1: There was no information on patient inclusion/exclusion. The proportions of each ROI class were precisely 40%, 40% and 20%, indicating a heavy selection step so bias in the selection of frames and ROIs cannot be excluded. A test set of 30 samples from 10 pullbacks sounds very selective too, though it only appeared in the Introduction.  R2: While 31 patients were used, it is unclear whether the selection of frames was done randomly or if certain frames were excluded due to poor quality. This could introduce selection bias, especially given the manual segmentation performed by experts. |  |
| B. Applicability | |
| **Concern that the included participants and setting do not match the review question** **(low/high/unclear)** | **High** |
| *Rationale of applicability rating:*  R1 & R2: No patient inclusion/exclusion criteria or demographics were provided, which limits the applicability to routine imaging. The concern was further raised by the inconsistencies as described earlier. |  |

| Title | |
| --- | --- |
| 1243. In vivo detection of plaque erosion by intravascular optical coherence tomography using artificial intelligence | |
| Description of Data (source, inclusion/exclusion, setting and dates, etc.) | |
| 83 OCT pullbacks were collected from 83 ACS patients with culprit plaque erosion who had undergone pre-intervention OCT imaging of culprit lesions. All data were acquired at the Second Affiliated Hospital of Harbin Medical University using Abbott system.  A total of 29,914 frames with 3275 definite erosions. Probable erosions were not assessed in the study design but elaborated in the paper. Training-validation-testing split of 3:1:1 at the pullback level. | |
| Domain 1: Participants | |
| Signalling Questions | |
| 1.1 Were appropriate data sources used, e.g. cohort, RCT or nested case-control study data? | Unclear |
| 1.2 Were all inclusions and exclusions of participants appropriate? | Unclear |
| A. Risk of Bias | |
| **Risk of bias with participants selection (low/high/unclear)** | **Unclear** |
| *Rationale of bias rating:*  R1: Participants were ACS patients with culprit plaque erosion. No other information on inclusion/exclusion and demographics was given. Focusing on only ACS patients may create bias as erosions without ACS were overlooked, but the exact influence is unclear.  R2: The paper presents a clear selection of participants, focusing on patients with confirmed plaque erosion, which minimizes the risk of selection bias. However, the lack of detailed information regarding exclusion criteria, particularly how they handled stent-related cases or severe calcification, could introduce a potential bias. |  |
| B. Applicability | |
| **Concern that the included participants and setting do not match the review question** **(low/high/unclear)** | **High** |
| *Rationale of applicability rating:*  R1: As discussed, there was not enough information on patient inclusion/exclusion and demographics. Focusing on ACS patients only is sufficient for the original research question, but may limit its applicability to the routine diagnosis/prognosis.  R2: The model appears to be developed specifically for plaque erosion detection in patients with acute coronary syndrome, which directly aligns with clinical needs. However, the dataset used was confined to a single institution, limiting generalisability. Additionally, the study does not provide external validation, reducing its applicability to broader populations. |  |

| Title | |
| --- | --- |
| 4875. Structure attention co-training neural network for neovascularization segmentation in intravascular optical coherence tomography | |
| Description of Data (source, inclusion/exclusion, setting and dates, etc.) | |
| A total 1950 frames from 70 pullbacks, acquired using Abbott system at the Department of Cardiology, Chinese PLA General Hospital, China. Patient inclusion/exclusion criteria can be found at NCT04220437. It should be noted that the all the participants were in-stent restenosis patients.  No other demographic information was given. Neovascularization segmented by 2 independent observers. Used 1273 frames from 44 patients for training, 379 from 11 patients for validation and 298 frames from 15 patients for testing. The paper did not mention the proportion of positive samples. | |
| Domain 1: Participants | |
| Signalling Questions | |
| 1.1 Were appropriate data sources used, e.g. cohort, RCT or nested case-control study data? | Low |
| 1.2 Were all inclusions and exclusions of participants appropriate? | High |
| A. Risk of Bias | |
| **Risk of bias with participants selection (low/high/unclear)** | **High** |
| *Rationale of bias rating:*  R1: Although the pullbacks were collected from an observational trial, all the participants were in-stent restenosis patients. This should have been clearly stated in the paper. ISR as an inclusion criterion led to a highly selective cohort corresponding to a small proportion of neovascularization, which may introduce selection bias. Moreover, if only images with NV were used, the data might not be representative enough.  There was no other information on the patient demographics.  R2: The dataset in this study includes a relatively small number of pullbacks (70 pullbacks with 1950 2D slices). Given the nature of IVOCT imaging, this may not provide enough variation in data, potentially leading to biased model performance. Additionally, there is no clear indication of inclusion or exclusion criteria, making it difficult to assess if patient selection could introduce bias. |  |
| B. Applicability | |
| **Concern that the included participants and setting do not match the review question** **(low/high/unclear)** | **High** |
| *Rationale of applicability rating:*  R1: As discussed earlier there was no patient demographic information which may limit the applicability. All the participants were patients with in-stent restenosis, and there is a high risk in applicability on routine diagnosis/prognosis, where stent was not present or neovascularization occurred at other sites.  R2: The study focuses on neovascularization segmentation, which is a specific task within the broader context of IVOCT imaging for coronary artery disease. The highly specific nature of the task could limit the general applicability of the model to broader clinical contexts. |  |

| Title | |
| --- | --- |
| 1376. Comprehensive Assessment of Coronary Calcification in Intravascular OCT Using a Spatial-Temporal Encoder-Decoder Network | |
| Description of Data (source, inclusion/exclusion, setting and dates, etc.) | |
| Data was collected using an Abbott system at Department of Cardiology, The Second Affiliated Hospital of Harbin Medical University, Harbin, China. 13844 frames (2627 with calcification) from 45 pullbacks of 45 patients were included. 2/45 were with stents. Data split at pullback level with 27 for training, 9 for validation and 9 for testing. There were additional 4 pullbacks with more severe calcification as further benchmark. No patient level information was given. Ground truth was obtained by consensus of two readers with a senior expert as arbitrator. | |
| Domain 1: Participants | |
| Signalling Questions | |
| 1.1 Were appropriate data sources used, e.g. cohort, RCT or nested case-control study data? | Unclear |
| 1.2 Were all inclusions and exclusions of participants appropriate? | Unclear |
| A. Risk of Bias | |
| **Risk of bias with participants selection (low/high/unclear)** | **Unclear** |
| *Rationale of bias rating:*  R1: As described, there was no inclusion/exclusion or demographical information in the manuscript. The proportion of calcified frames seem representative, but risk of bias may still exist.  R2: The paper does not sufficiently detail the inclusion and exclusion criteria for patient selection, and it is unclear how the sample size was determined. |  |
| B. Applicability | |
| **Concern that the included participants and setting do not match the review question** **(low/high/unclear)** | **Unclear** |
| *Rationale of applicability rating:*  R1: There was no information on patient inclusion/exclusion or demographics, which raised concerns on applicability. On the other hand, besides the standard test set, the study also tested the performance on additional data with more severe calcification, benchmarking the generalisability of the algorithm. Distribution of calcium area, arc, depth, thickness and volume was partially documented. This helps readers and potential users understand and evaluate the domain shift.  R2: While the methodology appears sound, the focus on a specific population without clear demographic diversity limits its generalisability. The results might not apply to different clinical environments or broader patient populations. |  |

| Title | |
| --- | --- |
| 4937. Semantic Segmentation of Atherosclerosis in Superficial Layer of IVOCT Images Using Deep Learning | |
| Description of Data (source, inclusion/exclusion, setting and dates, etc.) | |
| Image was collected at the Department of Cardiovascular Medicine, Wakayama Medical University. There was a total of 113 frames from which 5723 square patches were generated. Annotation was provided by a single reader. An 80-10-10 split was used for training-validation-testing. | |
| Domain 1: Participants | |
| Signalling Questions | |
| 1.1 Were appropriate data sources used, e.g. cohort, RCT or nested case-control study data? | High |
| 1.2 Were all inclusions and exclusions of participants appropriate? | Unclear |
| A. Risk of Bias | |
| **Risk of bias with participants selection (low/high/unclear)** | **High** |
| *Rationale of bias rating:*  R1: There were only 113 frames, and the article did not mention how many pullbacks or patients were included. There was no information on inclusion/exclusion and demographics either. Given the small number of frames, the dataset seems rather selective, and the number of participants was likely to be very limited, both leading to a high risk in participants selection.  R2: The study provides limited information on participant selection and dataset diversity. The dataset is composed of 113 IVOCT images from only three patients, which is insufficient to ensure representativeness. |  |
| B. Applicability | |
| **Concern that the included participants and setting do not match the review question** **(low/high/unclear)** | **High** |
| *Rationale of applicability rating:*  R1: As described it was a very small cohort without information on the number of participants, inclusion/exclusion criteria or demographics. The applicability of both steps (ROI delineation using level-set and pixel-wise segmentation using SegNet) are likely to be affected.  R2: While the segmentation method is promising, the applicability of the results is limited due to the small dataset and lack of external validation. The model was trained and tested on data from only a few patients, which reduces its generalisability to broader clinical settings. |  |

| Title | |
| --- | --- |
| 4595. Detection of thin-cap fibroatheroma in IVOCT images based on weakly supervised learning and domain knowledge | |
| Description of Data (source, inclusion/exclusion, setting and dates, etc.) | |
| Used the 2017 CCCV-IVOCT contest dataset. This involved 2000 frames in polar coordinates for training, among which were 1000 positive samples with TCFA and 1000 negative samples. Another 300 frames (198 TCFAs) were used for testing. No patient information was provided in CCCV-IVOCT. The authors mentioned splitting the data at the patient level but it is unclear if this information was available in CCCV-IVOCT. | |
| Domain 1: Participants | |
| Signalling Questions | |
| 1.1 Were appropriate data sources used, e.g. cohort, RCT or nested case-control study data? | Unclear |
| 1.2 Were all inclusions and exclusions of participants appropriate? | Unclear |
| A. Risk of Bias | |
| **Risk of bias with participants selection (low/high/unclear)** | **Unclear** |
| *Rationale of bias rating:*  R1 & R2: CCCV-IVOCT dataset without additional validation. The risk of bias due to frame/patient selection cannot be excluded. |  |
| B. Applicability | |
| **Concern that the included participants and setting do not match the review question** **(low/high/unclear)** | **High** |
| *Rationale of applicability rating:*  R1 & R2: No patient inclusion/exclusion criteria or demographics were provided in CCCV-IVOCT dataset. There is a high risk in the applicability and generalisability to routine scans. |  |

| Title | |
| --- | --- |
| 2219. Histopathology-Based Deep-Learning Predicts Atherosclerotic Lesions in Intravascular Imaging | |
| Description of Data (source, inclusion/exclusion, setting and dates, etc.) | |
| Two datasets were involved: (1) 62 frames from 7 autopsy specimen with matching histopathology with high-quality annotation (2) clinical dataset screened from OCT database at the German Heart Centre Munich (including all patients undergoing intravascular imaging with clinical indication for OCT during coronary angiography). A total of 222 frames from 51 patients were used for analysis. In case of stented vessels, proximal and distal regions outside the stent were used. Patient demographics included, but it was unclear how screening was performed so that only 222 frames were left. Both datasets were acquired using the Abbott system. | |
| Domain 1: Participants | |
| Signalling Questions | |
| 1.1 Were appropriate data sources used, e.g. cohort, RCT or nested case-control study data? | Unclear |
| 1.2 Were all inclusions and exclusions of participants appropriate? | Unclear |
| A. Risk of Bias | |
| **Risk of bias with participants selection (low/high/unclear)** | **Unclear** |
| *Rationale of bias rating:*  R1 & R2: The study benefitted from an autopsy dataset where high-quality ground truth was available. However, the cause of death was not declared, and it was unclear if the pullbacks were performed in heavily diseased or even culprit vessels, thus a risk of bias cannot be excluded. For the clinical dataset, although the patient demographic information was given, the inclusion/exclusion criteria used in screening was not clearly stated. A total of 222 frames seems highly selective and bias may exist. |  |
| B. Applicability | |
| **Concern that the included participants and setting do not match the review question** **(low/high/unclear)** | **High** |
| *Rationale of applicability rating:*  R1 & R2: For the reasons stated above it is unclear how representative these two datasets were, raising concerns in applicability and generalisability. |  |

| Title | |
| --- | --- |
| 1964. Automatic classification of a-lines in intravascular oct images using deep learning and estimation of attenuation coefficients | |
| Description of Data (source, inclusion/exclusion, setting and dates, etc.) | |
| 183 frames derived from 33 patients who underwent OCT imaging with the Abbott system. Imaging data was acquired at 3 centres: Hippokration Hospital, Athens, Greece, General Hospital of Nikaia, Piraeus, Greece and New Tokyo Hospital, Chiba, Japan. Images were likely to be annotated by a single reader. The ground truth definition is unclear, out of the 183 frames there were 84 lipid, 80 calcified, 70 fibrous and 42 mixed plaques – perhaps the authors meant continuous A-line regions rather than the conventional definition of plaques. | |
| Domain 1: Participants | |
| Signalling Questions | |
| 1.1 Were appropriate data sources used, e.g. cohort, RCT or nested case-control study data? | Unclear |
| 1.2 Were all inclusions and exclusions of participants appropriate? | Unclear |
| A. Risk of Bias | |
| **Risk of bias with participants selection (low/high/unclear)** | **Unclear** |
| *Rationale of bias rating:*  R1 & R2: There was no description of patient inclusion/exclusion or demographics. 183 frames from 3 international centres seem rather selective and bias may exist, but it is difficult to assess the risk with the information available. |  |
| B. Applicability | |
| **Concern that the included participants and setting do not match the review question** **(low/high/unclear)** | **High** |
| *Rationale of applicability rating:*  R1: As discussed above, the lack of patient inclusion/exclusion and demographics may raise concerns on the applicability to routine diagnosis/prognosis. The frame selection and the resulting small sample size further increased the risk.  R2: The dataset size (183 images from 33 patients) limits the generalisability of the findings. |  |

| Title | |
| --- | --- |
| 1797. Identification of coronary calcifications in optical coherence tomography imaging using deep learning | |
| Description of Data (source, inclusion/exclusion, setting and dates, etc.) | |
| Initial cohort involved 8000 OCT images from consecutive patients that underwent coronary angiography with OCT at Soroka Medical Centre, Beer Sheva, Israel. There was no information on the number of pullbacks/patients or demographics. The initial cohort was assessed by two experts for calcification. Corrupted frames and frames with equivocal findings were dropped. Only frames with confirmed calcification were used for training. The final cohort reduced from 8000 to 540 frames only, with 490 used for training and 50 for testing. | |
| Domain 1: Participants | |
| Signalling Questions | |
| 1.1 Were appropriate data sources used, e.g. cohort, RCT or nested case-control study data? | Unclear |
| 1.2 Were all inclusions and exclusions of participants appropriate? | High |
| A. Risk of Bias | |
| **Risk of bias with participants selection (low/high/unclear)** | **High** |
| *Rationale of bias rating:*  R1: There was not enough patient-level information such as the number of participants and demographics. By dropping frames with equivocal findings, the algorithm was only trained and tested in ‘easier’ cases of calcification, creating a bias with sample selection.  R2: The paper lacks detailed information on participant inclusion and exclusion criteria. |  |
| B. Applicability | |
| **Concern that the included participants and setting do not match the review question** **(low/high/unclear)** | **High** |
| *Rationale of applicability rating:*  See the comments above. Furthermore, corrupted frames were dropped in the analysis, but corruption was not defined. Given the large number of frames being dropped (only 540/8000 remained), the corrupted frames are likely to include images with artefact, which are common in routine use. This may also raise concerns on the applicability on diagnosis/prognosis. |  |

| Title | |
| --- | --- |
| 1023. Automated diagnosis of optical coherence tomography imaging on plaque vulnerability and its relation to clinical outcomes in coronary artery disease | |
| Description of Data (source, inclusion/exclusion, setting and dates, etc.) | |
| In the TACUMI study, 46120 frames from 1791 patients (screened from 6625 patients from 2010 to 2019 at 3 university hospitals in Japan) with OCT imaging of non-culprit lesions were involved. There is a clear flow chart with all inclusion/exclusion criteria. Ground truth classification was given by 3 or 4 independent readers. 44947 images from 1689 patients used for model development with 80-20 training-validation split. Another 1173 frames from 102 patients from the same database were reserved for testing. Finally, 1450 patients in the development cohort completed the follow up to assess the prognostic value against clinical events. Demographics were given for the 1450 patients. | |
| Domain 1: Participants | |
| Signalling Questions | |
| 1.1 Were appropriate data sources used, e.g. cohort, RCT or nested case-control study data? | Low |
| 1.2 Were all inclusions and exclusions of participants appropriate? | Low |
| A. Risk of Bias | |
| **Risk of bias with participants selection (low/high/unclear)** | **Low** |
| *Rationale of bias rating:*  R1: A multi-centre study with a good number of participants, clear inclusion/exclusion criteria, patient demographic information and follow-up imaging. The only concern is that there were much fewer frames per patient in the test set which may introduce a selection bias.  R2: The study includes a large sample size (1791 patients), which reduces selection bias, but there are some concerns regarding exclusion criteria. The paper does not detail why some patients were excluded due to poor image quality or severe calcification, which could introduce bias by excluding harder-to-diagnose cases. |  |
| B. Applicability | |
| **Concern that the included participants and setting do not match the review question** **(low/high/unclear)** | **Unclear** |
| *Rationale of applicability rating:*  R1 & R2: As discussed above, the dataset of TACUMI study matched the review question. No immediate concerns of applicability on diagnostic or prognostic models developed on this dataset. However, it is unclear whether the model will be applicable outside of a Japanese population. It is currently unknown whether ethnicity can be deduced from the OCT imaging. |  |

| Title | |
| --- | --- |
| 2578. Detection of optical coherence tomography-defined thin-cap fibroatheroma in the coronary artery using deep learning | |
| Description of Data (source, inclusion/exclusion, setting and dates, etc.) | |
| Between May 2010 and May 2016, 6,598 consecutive patients with stable and unstable angina underwent invasive coronary angiography at Asan Medical Centre, Seoul, South Korea. Preprocedural OCT data were obtained using Abbott system in 798 patients. All patients had at least one lesion with 30%-85% of angiographic stenosis. After exclusion of stenting and poor- quality scans, 45400 frames from 602 lesions in 602 patients were involved. Demographics were given. Ground truth by 2 independent readers blinded to patient information. 480 patients assigned for development under 5-fold CV, and the remaining 122 for testing. Another dataset of 65 patients imaged between Feb 2016 and Nov 2017 were used for further validation. | |
| Domain 1: Participants | |
| Signalling Questions | |
| 1.1 Were appropriate data sources used, e.g. cohort, RCT or nested case-control study data? | Low |
| 1.2 Were all inclusions and exclusions of participants appropriate? | Low |
| A. Risk of Bias | |
| **Risk of bias with participants selection (low/high/unclear)** | **Low** |
| *Rationale of bias rating:*  R1 & R2: Clear inclusion/exclusion criteria and patient demographics were given. In addition to model validation and testing, there was another non-overlapping validation dataset. The only weakness is perhaps being a single-centre study which may be subject to bias. |  |
| B. Applicability | |
| **Concern that the included participants and setting do not match the review question** **(low/high/unclear)** | **Low** |
| *Rationale of applicability rating:*  See above. |  |

| Title | |
| --- | --- |
| 1096. Enhanced Diagnosis of Plaque Erosion by Deep Learning in Patients with Acute Coronary Syndromes | |
| Description of Data (source, inclusion/exclusion, setting and dates, etc.) | |
| The study utilized data from two trials, PREDICTOR (NCT03479723) for development and EROSION (NCT02041650) for testing. 237,021 frames from 581 patients in PREDICTOR with 80-20 split for model development. 65,394 frames from 292 patients in EROSION used for testing. Both datasets used the Abbott system. Patient demographics were given and there is a 60-page supplementary document detailing the inclusion/exclusion criteria and protocols. Care was given to avoid data overlapping and ensure each patient only contributed one exam. | |
| Domain 1: Participants | |
| Signalling Questions | |
| 1.1 Were appropriate data sources used, e.g. cohort, RCT or nested case-control study data? | Low |
| 1.2 Were all inclusions and exclusions of participants appropriate? | Low |
| A. Risk of Bias | |
| **Risk of bias with participants selection (low/high/unclear)** | **Low** |
| *Rationale of bias rating:*  R1 & R2: Data from a major multi-centre trial for model development and a single-centre trial for testing. Both datasets seem appropriate. Trial and patient-level information was sufficiently provided in the paper. |  |
| B. Applicability | |
| **Concern that the included participants and setting do not match the review question** **(low/high/unclear)** | **Low** |
| *Rationale of applicability rating:*  R1 & R2: There is enough data variance and information on data distribution. No immediate applicability concerns arising from study participants. |  |

| Title | |
| --- | --- |
| 4916. A Preliminary Study of IVOCT-Based Atherosclerosis Plaque Classification Technique | |
| Description of Data (source, inclusion/exclusion, setting and dates, etc.) | |
| Three IVOCT pullbacks, each containing approximately 370 frames, were obtained from University of Malaya Medical Centre (UMMC). The pullbacks contain frames of coronary arteries with fibrous and fibrocalcific plaque. The study involved 50 frames from 3 pullbacks, with 30 frames for training and 20 for testing. Ground truth annotation likely done by a single reader. | |
| Domain 1: Participants | |
| Signalling Questions | |
| 1.1 Were appropriate data sources used, e.g. cohort, RCT or nested case-control study data? | Unclear |
| 1.2 Were all inclusions and exclusions of participants appropriate? | High |
| A. Risk of Bias | |
| **Risk of bias with participants selection (low/high/unclear)** | **High** |
| *Rationale of bias rating:*  R1: There were only 3 pullbacks from which 50 frames were selected. There was no information at patient level. Only 50 frames were selected which are likely to introduce bias. Moreover, the pullbacks consisted of only fibrous and fibrocalcific plaque. This is not representative and further added the bias in samples.  R2: The study did not provide enough detail regarding how participants were selected, specifically how the inclusion and exclusion criteria were determined. |  |
| B. Applicability | |
| **Concern that the included participants and setting do not match the review question** **(low/high/unclear)** | **High** |
| *Rationale of applicability rating:*  See the discussion above. |  |

| Title | |
| --- | --- |
| 3884. Classification of calcium in intravascular OCT images for the purpose of intervention planning | |
| Description of Data (source, inclusion/exclusion, setting and dates, etc.) | |
| 35 pullbacks of LAD or LCX acquired prior to stenting were selected from the database available at the Cardiovascular Core Lab of University Hospitals Case Medical Centre (Cleveland). No information on patient selection and demographics. Frames were manually analysed by a single reader from the Core Lab to identify calcium, lipid, and fibrous plaques. 1566 sub-images (316 calcium-only, 250 lipid-only, 250 fibrous-only and 750 without component) were generated and used for development under 5-fold CV. An independent post-mortem pullback of 509 sub-images (14 were calcium) were used for testing. | |
| Domain 1: Participants | |
| Signalling Questions | |
| 1.1 Were appropriate data sources used, e.g. cohort, RCT or nested case-control study data? | Unclear |
| 1.2 Were all inclusions and exclusions of participants appropriate? | High |
| A. Risk of Bias | |
| **Risk of bias with participants selection (low/high/unclear)** | **High** |
| *Rationale of bias rating:*  R1 & R2: There was no information on patient selection or demographics. Working with sub-images with single component only may lead to both bias and variance issues. |  |
| B. Applicability | |
| **Concern that the included participants and setting do not match the review question** **(low/high/unclear)** | **High** |
| *Rationale of applicability rating:*  R1: The lack of patient level information, such as selection and demographics, may limit the applicability of the work. The algorithm was developed with sub-images of a single tissue type, which raised concerns on its applicability to routine scans that are highly heterogeneous. There were very few positive samples in the test set (14/509) and couldn’t assess the generalisability well. |  |

| Title | |
| --- | --- |
| 5374. Deep neural networks for A-line-based plaque classification in coronary intravascular optical coherence tomography images | |
| Description of Data (source, inclusion/exclusion, setting and dates, etc.) | |
| Total 4469 frames from 48 pullbacks of 48 patients, a subset from the TRANSFORM trial (NCT01972022). Ground truth annotation by consensus of two readers as either fibrocalcific, fibrolipidic or others. Data split of training, validation and testing was done on patient level. | |
| Domain 1: Participants | |
| Signalling Questions | |
| 1.1 Were appropriate data sources used, e.g. cohort, RCT or nested case-control study data? | Low |
| 1.2 Were all inclusions and exclusions of participants appropriate? | Unclear |
| A. Risk of Bias | |
| **Risk of bias with participants selection (low/high/unclear)** | **Unclear** |
| *Rationale of bias rating:*  R1 & R2: The dataset was a subset from the TRANSFORM trial. Although the inclusion/exclusion criteria and demographics were well documented in the original trial, it was unclear how the subset was selected in this study. The demographic distribution of this subset is also unknown. |  |
| B. Applicability | |
| **Concern that the included participants and setting do not match the review question** **(low/high/unclear)** | **Unclear** |
| *Rationale of applicability rating:*  As noted above, data was a subset from a well-documented trial but the criteria for selecting the subset was unknown. There was not enough patient-level information to assess the applicability either. |  |

| Title | |
| --- | --- |
| 3025. Coronary calcification segmentation in intravascular OCT images using deep learning: Application to calcification scoring | |
| Description of Data (source, inclusion/exclusion, setting and dates, etc.) | |
| The dataset included 48 VOIs taken from 34 clinical pullbacks using the Abbott system, giving a total of 2640 frames. The pullbacks are subset of the University Hospitals Cleveland Medical Centre (UHCMC) imaging library. Although the selection criteria and demographics were described the original study, it was unclear how this subset was selected. There was no demographical information of the subset either. Ground truth was annotated by two independent readers. There was no information on the proportion of positive VOIs or frames. VOIs not containing a calcification were also included. 80-10-10 VOI split for training, validation and testing. | |
| Domain 1: Participants | |
| Signalling Questions | |
| 1.1 Were appropriate data sources used, e.g. cohort, RCT or nested case-control study data? | Low |
| 1.2 Were all inclusions and exclusions of participants appropriate? | High |
| A. Risk of Bias | |
| **Risk of bias with participants selection (low/high/unclear)** | **High** |
| *Rationale of bias rating:*  R1: Data was a subset of a published prospective study. However, we do not know the criteria of subset selection or the patient demographics in the subset. The authors did not describe the proportion of positive samples which led to a high risk of bias.  R2: The study includes detailed information about how data were acquired, including pullbacks from 34 clinical cases, yet it lacks clarity on the specific criteria for participant selection. |  |
| B. Applicability | |
| **Concern that the included participants and setting do not match the review question** **(low/high/unclear)** | **High** |
| *Rationale of applicability rating:*  R1: As noted above, data was a subset from a well-documented trial but the criteria for selecting the subset was unknown. There was not enough patient-level information and we do not know the proportion of positive samples in the dataset, raising concerns on the applicability and generalisability.  R2: The methods used in this study, including segmentation and calcification scoring, align well with clinical needs, especially for stent planning in PCI. However, since the study used manually annotated datasets, the generalisability of the model to other datasets or real-world clinical settings remains uncertain. |  |

| Title | |
| --- | --- |
| 2973. Automated plaque characterization using deep learning on coronary intravascular optical coherence tomographic images | |
| Description of Data (source, inclusion/exclusion, setting and dates, etc.) | |
| The dataset consisted of 57 pullbacks from 55 patients with 89 VOIs (32 with calcification, 36 with lipid, 12 with both and 9 with neither), resulting in 4,892 frames. The images were acquired using the Abbott system. No information on patient selection or demographics was given. Annotations were performed by two independent readers. Data splitting was done at VOI level with an 80-10-10 ratio for nested CV. | |
| Domain 1: Participants | |
| Signalling Questions | |
| 1.1 Were appropriate data sources used, e.g. cohort, RCT or nested case-control study data? | Unclear |
| 1.2 Were all inclusions and exclusions of participants appropriate? | Unclear |
| A. Risk of Bias | |
| **Risk of bias with participants selection (low/high/unclear)** | **Unclear** |
| *Rationale of bias rating:*  R1 & R2: As described, there was not enough information on patient inclusion/exclusion criteria and their demographics. It is therefore difficult to assess the risk of bias with participants. |  |
| B. Applicability | |
| **Concern that the included participants and setting do not match the review question** **(low/high/unclear)** | **High** |
| *Rationale of applicability rating:*  R1: Due to the lack of information on patient selection and demographic characteristics, there are concerns on the model applicability and generalisability.  R2: The model was trained on data that might not represent the broad spectrum of clinical cases. |  |

| Title | |
| --- | --- |
| 2491. Fully automated plaque characterization in intravascular OCT images using hybrid convolutional and lumen morphology features | |
| Description of Data (source, inclusion/exclusion, setting and dates, etc.) | |
| The dataset consisted of pullbacks from 49 patients with 111 VOIs (34 with calcification, 37 with lipid, 13 with both and 27 with neither), resulting in 6,556 frames. The images were acquired using the Abbott system, and those with stent or poor quality were excluded. No information on patient selection or demographics was given. Annotations were performed by two independent readers. Data splitting was done at VOI level with an 3-1-1 ratio for nested CV. | |
| Domain 1: Participants | |
| Signalling Questions | |
| 1.1 Were appropriate data sources used, e.g. cohort, RCT or nested case-control study data? | Unclear |
| 1.2 Were all inclusions and exclusions of participants appropriate? | Unclear |
| A. Risk of Bias | |
| **Risk of bias with participants selection (low/high/unclear)** | **Unclear** |
| *Rationale of bias rating:*  R1 & R2: As described, there was not enough information on patient inclusion/exclusion criteria and their demographics. It is therefore difficult to assess the risk of bias with participants. |  |
| B. Applicability | |
| **Concern that the included participants and setting do not match the review question** **(low/high/unclear)** | **High** |
| *Rationale of applicability rating:*  R1 & R2: Due to the lack of information on patient selection and demographic characteristics, there are concerns on the model applicability and generalisability. |  |

| Title | |
| --- | --- |
| 2901. Automatic A-line coronary plaque classification using combined deep learning and textural features in intravascular OCT images | |
| Description of Data (source, inclusion/exclusion, setting and dates, etc.) | |
| The dataset consisted of 48 pullbacks with 80 lesion VOIs (32 with calcification, 36 with lipid, 12 with both), resulting in 4,292 frames. The images were acquired using the Abbott system. No information on patient selection or demographics was given. Annotations were performed by two independent readers. The data split is unclear. A 5-fold CV was used, and there was no description of a held-out set in Method. However, the flow chart talked about training and testing split without giving the proportions. Figure 5 mentioned a ‘test fold data of 8 VOIs (700 frames)’ but the numbers does not correspond to the overall dataset. Also 111 VOIs (same as in article 18) rather than 80 were reported in the accompanying presentation. | |
| Domain 1: Participants | |
| Signalling Questions | |
| 1.1 Were appropriate data sources used, e.g. cohort, RCT or nested case-control study data? | Unclear |
| 1.2 Were all inclusions and exclusions of participants appropriate? | Unclear |
| A. Risk of Bias | |
| **Risk of bias with participants selection (low/high/unclear)** | **Unclear** |
| *Rationale of bias rating:*  R1 & R2: As described, there was not enough information on patient inclusion/exclusion criteria and their demographics. It is therefore difficult to assess the risk of bias with participants. |  |
| B. Applicability | |
| **Concern that the included participants and setting do not match the review question** **(low/high/unclear)** | **High** |
| *Rationale of applicability rating:*  R1: Due to the lack of information on patient selection and demographic characteristics, there are concerns on the model applicability and generalisability. There seems to be conflicting information on the total VOIs of the dataset, which was likely to be a result of version changes but further raised out concerns.  R2: While the study demonstrates the potential for combining deep learning and textural features for plaque classification, the results are limited by the small sample size and lack of external validation. |  |

| Title | |
| --- | --- |
| 2801. Segmentation of Coronary Calcified Plaque in Intravascular OCT Images Using a Two-Step Deep Learning Approach | |
| Description of Data (source, inclusion/exclusion, setting and dates, etc.) | |
| The clinical dataset consisted of 8,231 frames from 68 vessels of 68 patients. The images were acquired using the Abbott system. Frames with poor quality due to luminal blood, unclear lumen, artifact, or reverberation were excluded. 4,335 frames from 47 lesions were found with calcification. No patient selection criteria or demographics were given. Annotations were performed by 2 readers, but it was unclear if the readings were independent. The methods described both a 5-fold CV with 80-10-10 split. The model was further tested in an ex-vivo cohort of 4320 frames from 2 repetitive scans of 4 vessels from 4 hearts. | |
| Domain 1: Participants | |
| Signalling Questions | |
| 1.1 Were appropriate data sources used, e.g. cohort, RCT or nested case-control study data? | Unclear |
| 1.2 Were all inclusions and exclusions of participants appropriate? | Unclear |
| A. Risk of Bias | |
| **Risk of bias with participants selection (low/high/unclear)** | **Unclear** |
| *Rationale of bias rating:*  R1 & R2: As described, there was not enough information on patient inclusion/exclusion criteria and their demographics. It is therefore difficult to assess the risk of bias with participants. |  |
| B. Applicability | |
| **Concern that the included participants and setting do not match the review question** **(low/high/unclear)** | **High** |
| *Rationale of applicability rating:*  R1 & R2: Due to the lack of information on patient selection and demographic characteristics, there are concerns on the model applicability and generalisability. |  |

| Title | |
| --- | --- |
| 2379. Learning with Fewer Images via Image Clustering: Application to Intravascular OCT Image Segmentation | |
| Description of Data (source, inclusion/exclusion, setting and dates, etc.) | |
| Total 3,741 frames from 60 VOIs in 41 pullbacks were involved in the study. Images were acquired using the Abbott System. No patient selection or demographic information was given. Calcium segmentations were performed by 2 readers, but it was unclear if the readings were independent. 26 VOIs were labelled as no clinically significant lesions and 34 were labelled as calcification. Approx 75% VOIs used for model development with 5-fold CV (3-1-1 split), and 25% as held out testing. Lumen/guidewire segmentation as pre-processing and calcium segmentation as main outcome. | |
| Domain 1: Participants | |
| Signalling Questions | |
| 1.1 Were appropriate data sources used, e.g. cohort, RCT or nested case-control study data? | Unclear |
| 1.2 Were all inclusions and exclusions of participants appropriate? | High |
| A. Risk of Bias | |
| **Risk of bias with participants selection (low/high/unclear)** | **High** |
| *Rationale of bias rating:*  R1 & R2: As described, there was not enough information on patient inclusion/exclusion criteria and their demographics. The VOIs involved in this study were either disease free or calcification which seem a biased selection. |  |
| B. Applicability | |
| **Concern that the included participants and setting do not match the review question** **(low/high/unclear)** | **High** |
| *Rationale of applicability rating:*  R1& R2: Due to the lack of information on patient selection and demographic characteristics, there are concerns on the model applicability and generalisability. Since VOIs were labelled as either disease free or calcification, the algorithm might not generalize well to the routine diagnosis/prognosis, where images are heterogenous with the presence of other tissue types. |  |

| Title | |
| --- | --- |
| 4728. Automated analysis of fibrous cap in intravascular optical coherence tomography images of coronary arteries | |
| Description of Data (source, inclusion/exclusion, setting and dates, etc.) | |
| 4,360 frames from 77 lesions in 41 pullbacks acquired using the Abbott System were involved in the study. Images were a subset of the TRANSFORM trial. Baseline patient demographics of the subset were shown. It was unclear how this subset was selected; however, the BMI (41 vs 23) and proportions of STEMI (46.6% vs 25.6%) were a lot higher than the complete TRANSFORM dataset. Annotation was generated by 2 experts, but it was unclear if performed independently. 2,363 frames were annotated as lipidic and 1997 as normal. A 90-10 split was used for development (3821 frames from 68 lesions) and held-out testing (539 frames from 9 lesions). Within the development cohort a 5-fold CV with 3-1-1 split was used. | |
| Domain 1: Participants | |
| Signalling Questions | |
| 1.1 Were appropriate data sources used, e.g. cohort, RCT or nested case-control study data? | Low |
| 1.2 Were all inclusions and exclusions of participants appropriate? | High |
| A. Risk of Bias | |
| **Risk of bias with participants selection (low/high/unclear)** | **High** |
| *Rationale of bias rating:*  R1 & R2: It was unclear how this subset was selected from TRANSFORM. The significant increase of BMI and STEMI proportions compared to the complete TRANSFORM dataset suggests a high risk of selection bias. |  |
| B. Applicability | |
| **Concern that the included participants and setting do not match the review question** **(low/high/unclear)** | **High** |
| *Rationale of applicability rating:*  R1 & R2: The BMI and STEMI rate were unusually high, making the dataset less representative of the routine imaging population. This led to concerns on the applicability and generalisability. |  |

| Title | |
| --- | --- |
| 4907. Automatic microchannel detection using deep learning in intravascular optical coherence tomography images | |
| Description of Data (source, inclusion/exclusion, setting and dates, etc.) | |
| 575 frames across 48 pullbacks imaged using the Abbott system were included in the study. 507 were used for development and 68 for testing. Abstract mentioned a total 3,075 frames across 41 patients with 62 microchannel segments, but this did not appear in the main text. The dataset is a subset of the TRANSFORM trial. Selection criteria and demographic information of the subset was not given. Ground-truth annotation (microchannel or other) was provided by one expert, and a second expert contributed to inter-observer agreement assessment. After 6x augmentation there were 3,450 frames, indicating that test images were also augmented. However, in Results the test set remained 68, suggesting a risk of data leaking into training or validation. | |
| Domain 1: Participants | |
| Signalling Questions | |
| 1.1 Were appropriate data sources used, e.g. cohort, RCT or nested case-control study data? | Low |
| 1.2 Were all inclusions and exclusions of participants appropriate? | High |
| A. Risk of Bias | |
| **Risk of bias with participants selection (low/high/unclear)** | **High** |
| *Rationale of bias rating:*  R1 & R2: There was no selection criteria or demographic information of the subset. It was unclear how the initial 3,075 and later 575 frames were selected. It was also unclear if all the 575 frames were found with microchannels. Therefore, there is a high risk with the bias in the inclusion and exclusion of samples. |  |
| B. Applicability | |
| **Concern that the included participants and setting do not match the review question** **(low/high/unclear)** | **High** |
| *Rationale of applicability rating:*  R1 & R2: There were no selection criteria mentioned for the subset. Given 62 microchannel segments were found in total, it was unclear if microchannels were present in all 575 frames used in the study. |  |

| Title | |
| --- | --- |
| 1075. Automated Segmentation of Microvessels in Intravascular OCT Images Using Deep Learning | |
| Description of Data (source, inclusion/exclusion, setting and dates, etc.) | |
| 8403 frames from 85 lesions and 37 normal segments were extracted from Abbott OCT of 79 patients. This is a subset of the TRANSFORM cohort. No information on the patient subset selection but it already covered 79/90 of the complete cohort. It was unclear how the 85 segments were selected given all 79 patients had multivessel disease. Ground-truth annotation (microchannel or other) was provided by one expert, and a second expert contributed to inter-observer agreement assessment. The proportion of positive samples was only reported in a sub-analysis of 60 ROIs, with 730 out of 2812 frames showing microvessel. It was unclear how these 60 ROIs were chosen and what was the proportion in the complete cohort. | |
| Domain 1: Participants | |
| Signalling Questions | |
| 1.1 Were appropriate data sources used, e.g. cohort, RCT or nested case-control study data? | Low |
| 1.2 Were all inclusions and exclusions of participants appropriate? | High |
| A. Risk of Bias | |
| **Risk of bias with participants selection (low/high/unclear)** | **High** |
| *Rationale of bias rating:*  R1: This study covered most of TRANSFORM patients, but the patient selection criteria and demographics was still missing. More importantly, there was no information on the segment selection or the proportion of positive samples in the analysis cohort. The proportion was only reported in a sub-analysis of 60 ROIs, but we do not know how the ROIs were further selected.  R2: The paper does not provide enough detailed information regarding the inclusion and exclusion criteria for participants or how the selection was made. While the dataset includes a large number of frames from various patients, it is unclear whether there was any sampling bias or how representative the sample was of the broader population. |  |
| B. Applicability | |
| **Concern that the included participants and setting do not match the review question** **(low/high/unclear)** | **High** |
| *Rationale of applicability rating:*  R1 & R2: The readers could not find the selection criteria for the segments and distribution of positive/negative samples in the population. This raised concerns in the applicability and generalisability of the work. |  |

| Title | |
| --- | --- |
| 159. Diagnosis of coronary layered plaque by deep learning | |
| Description of Data (source, inclusion/exclusion, setting and dates, etc.) | |
| Same group and dataset as article 6. The study utilized data from two trials, PREDICTOR (NCT03479723) for development and EROSION (NCT02041650) for testing. 237,021 frames from 581 patients in PREDICTOR with 5-fold CV for model development. 65,394 frames from 292 patients in EROSION used for testing. Both datasets used the Abbott system. Patient demographics were given in the main text; selection flow chart and subjects by centre were given in the supplementary material. The proportion of positive samples was also given for each data partition. | |
| Domain 1: Participants | |
| Signalling Questions | |
| 1.1 Were appropriate data sources used, e.g. cohort, RCT or nested case-control study data? | Low |
| 1.2 Were all inclusions and exclusions of participants appropriate? | Low |
| A. Risk of Bias | |
| **Risk of bias with participants selection (low/high/unclear)** | **Low** |
| *Rationale of bias rating:*  R1: Data from a major multi-centre trial for model development and a single-centre trial for testing. Both datasets seem appropriate. Trial and patient-level information was sufficiently provided in the paper. The proportion of positive samples was also given and seem sufficient.  R2: The study included a large, diverse, and well-curated dataset sourced from multiple institutions across four countries, significantly reducing the likelihood of selection bias. |  |
| B. Applicability | |
| **Concern that the included participants and setting do not match the review question** **(low/high/unclear)** | **Low** |
| *Rationale of applicability rating:*  R1: There is enough data variance and information on data distribution. No immediate applicability concerns arising from study participants.  R2: The study demonstrates strong applicability due to the large-scale dataset and external validation. |  |

| Title | |
| --- | --- |
| 634. A Framework for Automated Quantification of Calcified Coronary Artery from Intravascular Optical Coherence Tomography Images | |
| Description of Data (source, inclusion/exclusion, setting and dates, etc.) | |
| The dataset consisted of 2000 frames from pullbacks of 60 patients who were potential candidates for intravascular lithotripsy (IVL, Shockwave, CA). 1800 frames from 50 pullbacks were used for training and 200 frames from 10 pullback for testing. Data was provided by Shockwave Medical Inc; the exact selection criteria and demographic information was not shown. | |
| Domain 1: Participants | |
| Signalling Questions | |
| 1.1 Were appropriate data sources used, e.g. cohort, RCT or nested case-control study data? | Unclear |
| 1.2 Were all inclusions and exclusions of participants appropriate? | High |
| A. Risk of Bias | |
| **Risk of bias with participants selection (low/high/unclear)** | **High** |
| *Rationale of bias rating:*  R1: All images were obtained from potential candidates for IVL. As the authors noted in the paper, these participants majorly calcium-heavy when compared to average patients. There was no information on patient selection criteria, demographics or the evaluated calcium burden (only the quantified errors were shown).  R2: The paper presents a relatively well-defined dataset consisting of 2000 image frames from 60 patients. However, the study does not provide detailed information regarding the inclusion or exclusion criteria for these patients. |  |
| B. Applicability | |
| **Concern that the included participants and setting do not match the review question** **(low/high/unclear)** | **High** |
| *Rationale of applicability rating:*  R1 & R2: As the entire cohort were potential IVL candidates and no further information on the participants was given, there are raised concerns on the applicability and generalisability of the work to routine diagnosis or prognosis. |  |

| Title | |
| --- | --- |
| 3819. Multiscale distribution preserving autoencoders for plaque detection in intravascular optical coherence tomography | |
| Description of Data (source, inclusion/exclusion, setting and dates, etc.) | |
| OCT images were acquired using the Abbott system at New York Presbyterian Hospital/Columbia University Medical School, NY, USA. There were 15 pullbacks with 12 for training and 3 for testing. 12 frames were randomly chosen from each vessel in the training cohort, resulting in 144 frames. Patches were then randomly extracted. 30 frames were selected from the remaining 3 pullbacks for testing. There was no information on the patient selection criteria or demographics. It was unclear how the ground-truth labels were generated or how many positive samples there were. | |
| Domain 1: Participants | |
| Signalling Questions | |
| 1.1 Were appropriate data sources used, e.g. cohort, RCT or nested case-control study data? | Unclear |
| 1.2 Were all inclusions and exclusions of participants appropriate? | High |
| A. Risk of Bias | |
| **Risk of bias with participants selection (low/high/unclear)** | **High** |
| *Rationale of bias rating:*  R1 & R2: As a short conference paper, there was no information on the patient selection criteria or demographics. We do not know how the images/patches were labelled or how many positive samples there were. Therefore, a high risk of bias with participants selection seem to exist. |  |
| B. Applicability | |
| **Concern that the included participants and setting do not match the review question** **(low/high/unclear)** | **High** |
| *Rationale of applicability rating:*  R1 & R2: For the reasons reported above, there are concerns over the generalisability and applicability of the work. |  |

| Title | |
| --- | --- |
| 5287. Automated A-line coronary plaque classification of intravascular optical coherence tomography images using handcrafted features and large datasets | |
| Description of Data (source, inclusion/exclusion, setting and dates, etc.) | |
| The in-vivo dataset consisted of 6,556 frames from 49 patient pullbacks. The ex-vivo dataset consisted of 440 frames from 10 segments of 10 pullbacks. The OCT images were acquired using the Abbott system, and ex-vivo OCTs were coregistered with cryo-images. 4,819 frames from 41 patients of in-vivo dataset and the entire ex-vivo dataset was used for training under 5-fold CV. The rest 1,737 frames from 22 segments from 8 patients were used for held-out testing. Patient-level split was used for both CV and train/test. A-lines were sampled to provide balanced classes.  No patient inclusion/exclusion or demographics were given. Patients in the held out set contributed 2x frames compared to those in training set. | |
| Domain 1: Participants | |
| Signalling Questions | |
| 1.1 Were appropriate data sources used, e.g. cohort, RCT or nested case-control study data? | Unclear |
| 1.2 Were all inclusions and exclusions of participants appropriate? | Unclear |
| A. Risk of Bias | |
| **Risk of bias with participants selection (low/high/unclear)** | **Unclear** |
| *Rationale of bias rating:*  R1& R2: There was no selection criteria or demographic information of the subset. The sampling of a-lines appears reasonable; however it was unclear why patients in the held-out set contributed twice as many frames as those in the training set. |  |
| B. Applicability | |
| **Concern that the included participants and setting do not match the review question** **(low/high/unclear)** | **High** |
| *Rationale of applicability rating:*  R1& R2: Due to the lack of information on patient selection and demographic characteristics, there are concerns on the model applicability and generalizability. |  |

| Title | |
| --- | --- |
| 4623. Multi-view Contour-constrained Transformer Network for Thin-cap Fibroatheroma Identification | |
| Description of Data (source, inclusion/exclusion, setting and dates, etc.) | |
| Two datasets were used. The first was CCCV-IVOCT (2000 frames for training and 300 for testing, no patient-level information). The second was an in-house dataset collected using an Abbott system at the Second Affiliated Hospital of Harbin Medical University between Jun 2015 to Aug 2016. There was a total of 6500 frames (resampled from 31049 for balancing) from 2137 vessel segments of 540 patients. The patients aged between 18 to 80 and were without stenting. No other patient information was provided. TCFA identification was performed by 2 readers with another senior expert for arbitration. | |
| Domain 1: Participants | |
| Signalling Questions | |
| 1.1 Were appropriate data sources used, e.g. cohort, RCT or nested case-control study data? | Low |
| 1.2 Were all inclusions and exclusions of participants appropriate? | Unclear |
| A. Risk of Bias | |
| **Risk of bias with participants selection (low/high/unclear)** | **Unclear** |
| *Rationale of bias rating:*  R1 & R2: This study involved two separated cohorts which reduced the risk of bias in participants selection. However, there was insufficient information on the patient inclusion/exclusion and demographics, so that overall risk of bias is still unclear. |  |
| B. Applicability | |
| **Concern that the included participants and setting do not match the review question** **(low/high/unclear)** | **Unclear** |
| *Rationale of applicability rating:*  R1 & R2: The use of two cohorts may lead to better generalizability. However, the applicability may still be compromised by the lack of patient- and cohort-level information. |  |

| Title | |
| --- | --- |
| Deep learning segmentation of fibrous cap in intravascular optical coherence tomography images | |
| Description of Data (source, inclusion/exclusion, setting and dates, etc.) | |
| Two datasets were used: 24209 images (15239 calcification and 8970 FC) from 153 pullbacks of 77 patients from the TRANSFORM trial as development cohort, and 8322 images (6960 calcification and 1362 FC) from 74 pullbacks of 74 patients from Cleveland local registry for held-out test. Inclusion/exclusion criteria were documented. | |
| Domain 1: Participants | |
| Signalling Questions | |
| 1.1 Were appropriate data sources used, e.g. cohort, RCT or nested case-control study data? | Low |
| 1.2 Were all inclusions and exclusions of participants appropriate? | Unclear |
| A. Risk of Bias | |
| **Risk of bias with participants selection (low/high/unclear)** | **Unclear** |
| *Rationale of bias rating:*  R1 & R2: This study utilized data from the TRANSFORM trial and a local registry. The TRANSFORM trial has been published and well documented, and this study involved most subjects in trial, therefore reducing the risk in patient selection. However, only images with calcification and FC were included, which can lead to insufficient variance and a risk of selection bias.  Five-fold CV with 60-20-20 splits at pullback level in each fold, so no concerns on inappropriate partition or data leakage. |  |
| B. Applicability | |
| **Concern that the included participants and setting do not match the review question** **(low/high/unclear)** | **Unclear** |
| *Rationale of applicability rating:*  R1 & R2: The use of two cohorts may lead to better generalizability. However, only calcium and FC images were included in the model development and held-out testing. It is therefore unclear how applicable the model is in complete routine pullbacks. |  |
| Title | |
| 45. PolarFormer: A Transformer-Based Method for Multi-Lesion Segmentation in Intravascular OCT | |
| Description of Data (source, inclusion/exclusion, setting and dates, etc.) | |
| After exclusion, 7988 frames from 70 pullbacks from 70 ACS patients between 2019 and 2022 at Cardiovascular and Cerebrovascular Research Center of Sichuan Provincial People’s Hospital were utilized. Exclusion criteria were poor blood clearance, stenting and catheters off lumen centre. The last criterion might be too stringent as it is commonly seen in clinical pullbacks and in most cases interpretable. No demographics information.  Multiple annotators for fibrous (4934), calcified (3553) plaques and TCFA (1662). It is unclear how healthy, AIT, PIT, and in particular, ThCFA frames were handled.  Pullback level partition for training (57), validation (7) and test (6). | |
| Domain 1: Participants | |
| Signalling Questions | |
| 1.1 Were appropriate data sources used, e.g. cohort, RCT or nested case-control study data? | Unclear |
| 1.2 Were all inclusions and exclusions of participants appropriate? | Unclear |
| A. Risk of Bias | |
| **Risk of bias with participants selection (low/high/unclear)** | **Unclear** |
| *Rationale of bias rating:*  R1 & R2: No demographic information was found. Exclusion of images where catheters were off-centre is too stringent and likely increase the risk of bias. Though not in the exclusion criteria, it appears that healthy, AIT, PIT and ThCFA classes were absent in the training, validation and testing of the model.  The authors mentioned that both images and source code will be released after publication, which may potentially allow better assessment of the risk. However, at the time of our review (7 months after publication) the GitHub link does not work. |  |
| B. Applicability | |
| **Concern that the included participants and setting do not match the review question** **(low/high/unclear)** | **High** |
| *Rationale of applicability rating:*  R1 & R2: Due to the reason discussed above, there is a high concern on the applicability of the model when applied to routine clinical OCT imaging. Due to the stiffness, OCT catheters are often found off-centre, especially at changes of luminal geometry due to plaque formation or vessel curvature. However, such images were removed from the current analysis. The missing classes, especially ThCFA in the annotation also led to increased concern. |  |

| Title | |
| --- | --- |
| 46. A transformer-based pyramid network for coronary calcified plaque segmentation in intravascular optical coherence tomography images | |
| Description of Data (source, inclusion/exclusion, setting and dates, etc.) | |
| The dataset consisted of 2151 calcium-containing frames from 62 pullbacks from potential candidates for intravascular lithotripsy (IVL, Shockwave, CA). 1545 frames from 44 pullbacks were used for training and 606 frames from 18 pullback for testing. Data was provided by Shockwave Medical Inc and is confidential; the exact selection criteria and demographic information was not shown. | |
| Domain 1: Participants | |
| Signalling Questions | |
| 1.1 Were appropriate data sources used, e.g. cohort, RCT or nested case-control study data? | Unclear |
| 1.2 Were all inclusions and exclusions of participants appropriate? | High |
| A. Risk of Bias | |
| **Risk of bias with participants selection (low/high/unclear)** | **High** |
| *Rationale of bias rating:*  R1 & R2: All images were obtained from potential candidates for IVL. As the authors noted in the paper, these participants majorly calcium-heavy when compared to average patients. There was no further information on patient selection criteria, demographics or the evaluated calcium burden. |  |
| B. Applicability | |
| **Concern that the included participants and setting do not match the review question** **(low/high/unclear)** | **Unclear** |
| *Rationale of applicability rating:*  R1 & R2: The dataset is diverse in terms of artefacts and geometry, which would improve its generalizability. On the other hand, as the entire cohort were potential IVL candidates and no further information on the participants was given, there are raised concerns on the applicability of the work to routine diagnosis or prognosis. |  |

| Title | |
| --- | --- |
| 47. Automated volumetric intravascular plaque classification using optical coherence tomography | |
| Description of Data (source, inclusion/exclusion, setting and dates, etc.) | |
| The dataset was selected from a large database of manually analyzed OCT images in clinical setting. In total, 287 images from 35 pullbacks (LAD or LCx) of 35 patients were studied. No inclusion/exclusion criteria or demographic information was given. 311 VOIs were annotated as one of the 3 plaque classes (fibrous, lipid, calcium). Ex-vivo cryo images (106 in total) were used for validation.  Features were extracted from pixels within the annotated VOI and fed into an SVM classifier. Hyperparameters (SVM regularization and RBF kernel bandwidth) were selected using internal 5-fold CV with grid search. Performance was evaluated using the average of LOOCV. | |
| Domain 1: Participants | |
| Signalling Questions | |
| 1.1 Were appropriate data sources used, e.g. cohort, RCT or nested case-control study data? | High |
| 1.2 Were all inclusions and exclusions of participants appropriate? | High |
| A. Risk of Bias | |
| **Risk of bias with participants selection (low/high/unclear)** | **High** |
| *Rationale of bias rating:*  R1: The work utilized a very small dataset (287 frames from 35 pullbacks) from a large database. Patient inclusion/exclusion criteria and demographic information were not given. Within the 35 pullbacks it was also unclear how the 287 frames were chosen (only ~8 frames per pullback). The classifier relied on pixel-wise features which were only extracted from within the annotated VOI. These limitations together raised the risk of bias with sample selection. |  |
| B. Applicability | |
| **Concern that the included participants and setting do not match the review question** **(low/high/unclear)** | **High** |
| *Rationale of applicability rating:*  R1: Please refer to the comments above. |  |
